# Potential of Nanonutraceuticals in Increasing Immunity

**DOI:** 10.3390/nano10112224

**Published:** 2020-11-09

**Authors:** Josef Jampilek, Katarina Kralova

**Affiliations:** 1 Department of Analytical Chemistry, Faculty of Natural Sciences, Comenius University, Ilkovicova 6, 842 15 Bratislava, Slovakia; 2 Regional Centre of Advanced Technologies and Materials, Faculty of Science, Palacky University, Slechtitelu 27, 783 71 Olomouc, Czech Republic; 3 Institute of Chemistry, Faculty of Natural Sciences, Comenius University, Ilkovicova 6, 842 15 Bratislava, Slovakia; kata.kralova@gmail.com

**Keywords:** antioxidants, bioactive agents, curcumin, dietary supplements, encapsulation, foods, immunity, minerals, nanoparticles, nanoformulations, nutraceuticals, omega-3 fatty acids, probiotics, vitamins

## Abstract

Nutraceuticals are defined as foods or their extracts that have a demonstrably positive effect on human health. According to the decision of the European Food Safety Authority, this positive effect, the so-called health claim, must be clearly demonstrated best by performed tests. Nutraceuticals include dietary supplements and functional foods. These special foods thus affect human health and can positively affect the immune system and strengthen it even in these turbulent times, when the human population is exposed to the COVID-19 pandemic. Many of these special foods are supplemented with nanoparticles of active substances or processed into nanoformulations. The benefits of nanoparticles in this case include enhanced bioavailability, controlled release, and increased stability. Lipid-based delivery systems and the encapsulation of nutraceuticals are mainly used for the enrichment of food products with these health-promoting compounds. This contribution summarizes the current state of the research and development of effective nanonutraceuticals influencing the body’s immune responses, such as vitamins (C, D, E, B_12_, folic acid), minerals (Zn, Fe, Se), antioxidants (carotenoids, coenzyme Q10, polyphenols, curcumin), omega-3 fatty acids, and probiotics.

## 1. Introduction

Immunity is the ability of an organism to defend itself (and be resistant) against antigens originating both from the external and its internal environment. An antigen is generally any molecule capable of eliciting an immune system response. In practice, these are mainly pathogenic organisms, viruses, or tumor cells. Immunity triggers an immune response against these antigen-characterized subjects. Three defensive lines can be distinguished with the first two being non-specific and the third one specific. The first line is external. It is represented by covering tissues (skin and mucous membranes and their secretions) and prevents free contact of harmful substances with the internal environment. The second line is the internal non-specific defense realized by phagocytic cell types (cellular immunity) and the so-called complement (protein-based substances, see below) in places where the body is attacked (homeostasis is violated). Inflammation occurs as a result of this non-specific defense. The third line, the so-called acquired immunity, is specific. It is usually triggered at the same time as the second line of defense and responds in a specific way to specific recognized microorganisms, cells, toxins, etc. This line of defense is realized by lymphocytes and antibodies [1,2,3,4].

Immunity is generally divided into innate (natural) and adaptive (acquired). Innate immunity provides a basic defense against infection and serves as a constant “vigilant” guardian in the event of a sudden attack on the body. The principle of this immunity is that each cell expresses various typical molecules (pathogen-associated molecular formulas), which are recognized by receptors on cells of the innate immune system, on its surface. Phagocytes are most often involved in innate immune responses. Adaptive immunity is activated at a later stage of the “fight”, when the presence of the “intruder” and its pathogenic molecules (called antigens) activates specific T cell and B cell clones through specific antigenic immunoreceptors, thereby producing specific antibodies against intruders with specific antigens. Acquired immunity has the ability to remember this reaction, and the future immune response is thus faster and stronger. This immunological memory is the essence of vaccination. Adaptive immunity is mainly mediated by lymphocytes: T cells are a part of cellular immunity; B cells are responsible for humoral immunity, producing specific antibodies. Thus, according to the nature of the immune response, immunity can be divided into cellular and humoral. Cellular immunity is provided by many types of white blood cells. Phagocytic types (macrophages or neutrophils) provide a non-specific defense. Lymphocytes, which are involved in adaptive immunity and look for and kill pathogens or tumor cells, are another type. These cells mature mainly in the bone marrow, and there are about 7.4 × 10^9^ cells per liter of blood. Bad functioning of white blood cells results in autoimmune disease or immunodeficiency. Humoral immunity (provided by protein-based substances) is part of both the innate and adaptive components of the immune system. This includes “a natural immune shield”, the complement that indicates foreign structures, kills pathogenic cells, or causes inflammation. However, antibodies produced by B-lymphocytes that specifically bind to the antigen (the surface of a microorganism, virus, or cancer cells) and subsequently induce its destruction are the basic component of adaptive humoral immunity. In addition, interferons, substances produced by some white blood cells, especially in response to a virus attack, are included to this type of immunity [1,2,3,4].

As mentioned above, immunity plays an irreplaceable role in maintaining health. In addition to hereditary predispositions and the influence of the environment and stress, also nutrition has a significant effect on the correct functioning of immunity. In general, there is an interaction between nutrition and immunity. Malnutrition reduces the immune defense (especially non-specific innate and cell-mediated immunity), leading to a higher susceptibility of an individual to infection. On the other hand, an excess of food consumption, which can lead to obesity, does not mean a qualitative improvement in immunity. Unfortunately, the opposite is true. There are studies that show that obese people have lower levels of vitamins A and D (and thus impaired carbohydrate and fat metabolism) as well as lower levels of water-soluble vitamins (full spectrum of B vitamins, folate, and vitamin C) [5,6]. On the other hand, obesity has an essential need for an excess of Zn, Mn, Mg, Cr, and V, i.e., micro- and trace elements that are part of the enzymes involved in the metabolism of carbohydrates and fats. At the same time, these people usually consume little plant-based food, which only exacerbates all these shortcomings and creates a vicious circle [7]. The proper functioning and setting of immunity thus depends on the diverse composition and quality of the diet. Optimal nutrition at the early stages of life (breastfeeding), including pregnancy, also plays a role [8]. An effective immune response requires optimal levels of micronutrients, essential amino acids, fatty acids, vitamins, and probiotic bacteria [5,6,7,8,9].

Nutraceuticals (the term combining the words “nutrition” and “pharmaceutical”) are defined as foods or their extracts that have a demonstrably positive effect on human health and are the response of Western society to the shortcomings of commonly consumed diets. The group of nutraceuticals includes food supplements, which contain concentrated active ingredients from a given food, and functional foods, which are defined as foods to which an active therapeutic substance is added so as to have an effect on an individual health. It is important to note that, based on a decision of the European Food Safety Authority (EFSA), only foods that have been labeled with health claims based on studies can be considered nutraceuticals [10,11,12,13,14,15,16,17]. Nutraceuticals include substances of various groups such as minerals, trace elements, vitamins, alkaloids, oligo- and polysaccharides, fiber, amino acids, substances of a proteinaceous nature, fatty acids, fats, and probiotics. All of these compounds act on various organs and systems of the human body, either directly (by supplementing essential or missing substances) or by interfering with cells/tissues. They most often act in the prevention of disease states or as supplement substances that are difficult to obtain from ordinary food. Thus, nutraceuticals can affect the immune, gastrointestinal, urogenital, cardiovascular, and central nervous systems as well as body weight and prevent hormonal disorders or premature aging [10,11,12,13,14,15].

Currently, the world’s population is facing an onslaught of coronavirus disease (COVID-19) caused by the SARS-CoV-2 virus. Because safe vaccination is not yet available and specific/effective antivirals are missing, it seems that proper nutrition is a key factor in maintaining good health. By comparison with other viral diseases, it can be assumed that sufficient levels of vitamins C, D, and E, minerals Zn and Se, omega-3 fatty acids, as well as the desirable modulation of the intestinal microflora by suitable probiotics are crucial for increasing immunity against viral infections. It has been shown that supplementation with these nutrients or the administration of probiotics can be a potentially good way to reduce morbidity and mortality in patients with COVID-19 [18,19,20,21,22,23,24,25,26].

One of the modern ways of delivering bioactive substances to humans and animals is nanoformulations. This type of formulation is mainly used for the delivery of highly effective drugs, such as cytostatics, because it allows the targeted distribution of drugs to the affected tissues and controlled release, thereby suppressing the negative effects of the drug [27,28,29,30]. In addition, the overall bioavailability of the drug in the body can be modified, and this innovative formulation method is often used to overcome drug resistance [27,30,31,32,33,34]. In addition to cytostatics, many other bioactive compounds and even food supplements and foods for special medical purposes are used as nanoformulations [35,36,37]. In these cases, nanoformulations are primarily used to affect biodegradability and controlled release and increase stability (prevent degradation).

At present, nanomedicine and nanopharmaceuticals are regulated according to guidelines and regulations, primarily by the European Medicines Agency and the U.S. Food and Drug Administration as well as the regulations of national authorities [38,39,40,41,42,43,44,45]. This is due to the fact that permeation through membranes and the stability of nanosystems are closely related to the particle size and shape of nanoparticles (NPs), which is closely connected with toxic effects of the NP (undesired permeation of NP into non-target organs and tissues, excessive stability and accumulation of carriers, etc.), because NPs, due to their size, are able to induce oxidative stress, inflammation, and DNA damage, which can lead to irreversible tissue damage and cell death as well as the fact that positively charged nanocarriers are considered more toxic than negatively charged NPs due to their high ability to interact and be uptaken by cells [28,32,34,35,46,47,48,49,50,51,52]. Therefore, pharmaceutical manufacturers and the food industry prefer to use non-toxic biodegradable biopolymers, mainly of natural origin, for the production of nanoformulations. The EFSA issued draft guidelines for risk assessment of nanotechnologies used in food and feed [53], and it would be desirable for the whole nanoformulation, even if it contains active ingredients, which are “harmless” and classified as food supplements, to be tested primarily in vivo for its possible organ toxicity due to its nanoscale size.

**Figure 1:**
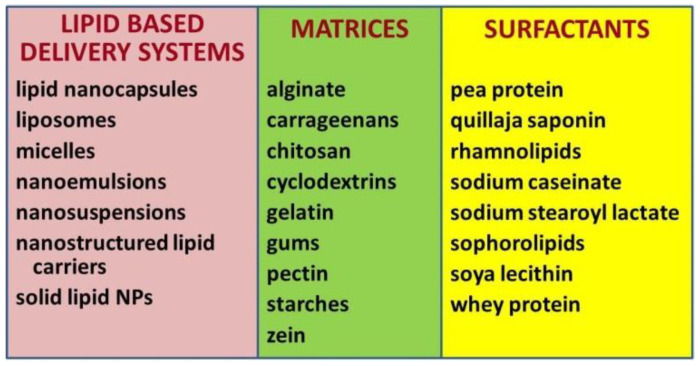
Nanoscale lipid-based delivery systems, matrices and surfactants most frequently used in nanoformulations of nutraceuticals.

The most common lipid-based delivery systems used by the pharmaceutical and food industry and agriculture include micelles, liposomes, nanoemulsions (NEs), nanosuspensions, lipid nanocapsules (LNCPs), solid lipid nanoparticles (SLNPs), nanostructured lipid carriers (NLCs), and lipid–polymer hybrid nanoparticles. For their formulation, non-toxic biodegradable polymers such as alginate (ALG), pectin and its modifications, carrageenans, gellan gum, xanthan gum, gum arabic (GA), hyaluronic acid, chitin, chitosan (CS), gelatin, cyclodextrins, cellulose and its semi-synthetic derivatives, as well as starches are usually used [27,37,46,47,48,54]. Nano- and microencapsulation techniques using these lipid-based carriers of bioactive agents were comprehensively reviewed previously [30,55,56,57,58,59,60,61,62,63,64]. The nanoscale lipid-based delivery systems, biopolymers and biosurfactants that are most frequently used in nanoformulations of nutraceuticals are presented in Figure 1.

As mentioned above, nutraceuticals include a wide range of substances with a significant effect on human immunity. However, this favorable impact can be enhanced by the fabrication of formulations containing additional biologically active components improving immunity. This paper provides a comprehensive, up-to-date overview of the findings related to important nutraceuticals, such as vitamins, minerals, antioxidants, omega-3 fatty acids, and probiotics, which are processed in nanoformulations, and their beneficial effects on immunity.

## 2. Vitamins

### 2.1. Vitamin C

**Figure 2:**
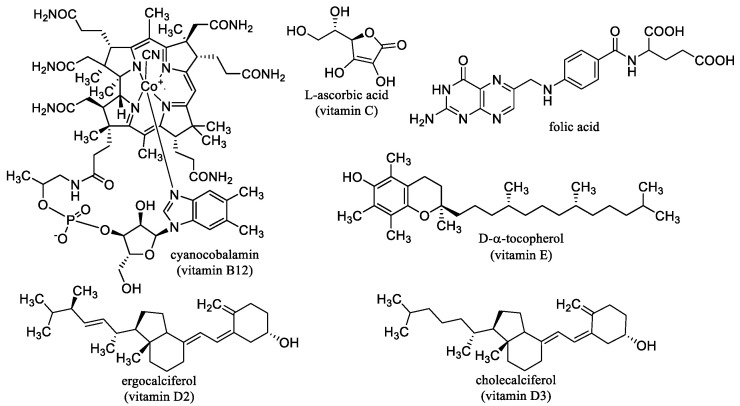
Structures of vitamins supporting immunity.

l-Ascorbic acid, vitamin C (Figure 2, food antioxidant E300) is a water-soluble compound necessary for life and maintaining physical health. It is sensitive to heat and light, highly sensitive to oxidation. Vitamin C was isolated in 1928 by the Hungarian biochemist, winner of the 1937 Nobel Prize in Physiology and Medicine Albert Szent-Györgyi. Most animals and plants are able to synthesize this vitamin; people have to eat it. Many fruits and vegetables are rich in vitamin C. The body does not build up reserves of vitamin C, the excess is excreted by the kidneys. During heat treatment of food there is a sharp loss of vitamin C. Vitamin C is needed for amino acid metabolism and collagen synthesis, so the deficiency is manifested by a decrease in blood vessel wall strength (increased bleeding) and reduced strength of the fibrous apparatus of the tooth (wobbling, tooth loss). Vitamin C is also important for tissue respiration. It supports the absorption of iron, stimulates the production of white blood cells, the development of bones, teeth and cartilage, and promotes growth. It also participates in the antioxidant defense of the cell (reduces the tocopheryl radical); on the other hand, under certain conditions, it has a prooxidative effect. The recommended daily dose of vitamin C is about 90 mg/day. Avitaminosis causes scurvy, anemia, bleeding, joint swelling, bone fragility, sterility, infections, atrophy, and stomach ulcers [65,66,67].

Vitamin C and folic acid (FA)-co-loaded liposomes (100–150 nm) were characterized with good stability and showed higher encapsulation efficiency (EE) and antioxidant activity than liposomes loaded with individual vitamins [68]. Vitamin C-encapsulated proliposome powder released 90% of vitamin C within 2 h, showed higher ex vivo antioxidant activity in brain and liver cells and a reduced malondialdehyde level of liver cells compared to free vitamin C [69]. The size of liposomes co-loaded with vitamin C and FA increased after coating with CS from 138 nm to 249 nm, and CS coating resulted in the improved physical stability as well as antioxidant effectiveness of co-loaded liposomes [70]. Sequential deposition of CS and sodium alginate (Na-ALG) onto the surface of anionic nanoliposomes affected liposomal membrane structure stability, provided a steric barrier on the surface resulting in a sustained release of encapsulated vitamin C, and protected it from oxidation and hydrolysis. Moreover, the fortification of mandarin juice with this formulation affected its organoleptic characteristics to a lesser extent than with naked nanoliposomes and free vitamin C, while higher microbiological stability was observed [71].

In an in vitro experiment, the hydrogels prepared by entrapment of Ca^2+^ with vitamin D or Fe^2+^ with vitamin C into a pectin/polyethylene glycol (PEG) polymer blend matrix showed only slow release of metal ions and vitamins in simulated gastric fluid (SGF) at pH 1.2 for 3 h, while in the next 3 h, when the hydrogels were situated in simulated intestinal fluid (SIF) at pH 6.8, high co-release of nutrients at the intestinal site was observed [72]. On the other hand, Salaheldin and Regheb [73] prepared Fe_3_O_4_ nanoparticles (NPs) coated with vitamin C, which ensured active absorption of iron “masked” as vitamin C in the body. In vivo studies in rats revealed that the administration of biscuits enriched with 10 ppm and 30 ppm nanoiron increased hemoglobin concentrations from 9.9 ± 1.2 g/dL to 14.6 ± 1.1 and 16.7 ± 1.6 g/dL, respectively [73].

As vitamin D has been described as a significant immune enhancer [74], it appears that the combination of vitamin C with vitamin D [72] may have the highest benefit of the above-mentioned nanoformulations for immunity.

### 2.2. Vitamin B_12_

The basis of the vitamin B_12_ molecule (Figure 2) is the complex bonding of the central cobalt atom with the four nitrogen atoms of the pyrrole nuclei, bound together in a corrin ring. Vitamin B_12_ is important especially for the proper function of hematopoiesis, involved in the synthesis of DNA and ATP, and essential for the proper function of the nervous system [67,75]. The main sources of this vitamin in the diet are eggs, milk, cheese, meat, and offal. It is not found in plant foods [67]. The proper supply of the necessary amount of vitamin B_12_ to the body improves memory, promotes concentration, and reduces the risk of heart disease. Vitamin B_12_ is part of preparations for the treatment of diseases of the liver, intestines, and pancreas. Its deficiency is manifested by pernicious anemia, weight loss, memory impairment, mental performance, muscle coordination, tremors and “tingling” in the limbs, and glossitis atrofica [67,75,76]. Vitamin B_12_ supply is problematic for vegetarians and vegans. Approx. 1.5–15% of the world’s population suffers from vitamin B_12_ deficiency. Risk factors contributing to vitamin B_12_ deficiency include veganism, atrophic gastritis, old age, gastrointestinal disease, alcoholism, anorexia, or malnutrition. Children breastfed by vegan mothers are also at risk [67,75,76].

Hydrogel based on *Auricularia polytricha* β-glucan showing a 3-D network with pore sizes of 30–300 µm which was characterized with a superb swelling ratio, pepsin stability, and pancrelipase-catalysed biodegradation property released 80% of encapsulated vitamin B_12_ in simulated intestinal fluid, while only <20% in SGF, suggesting that it can be used as intestinal target carrier for this vitamin [77]. Protein–lipid composite NPs showing a three-layered structure (a barley protein layer, α-tocopherol layer, and phospholipid layer) and an inner aqueous compartment, in which vitamin B_12_ was loaded, exhibited the controlled release of the vitamin in simulated gastrointestinal (GI) media. The oral administration of this nanoformulation to rats resulted in increased serum vitamin B_12_ levels and the reduced levels of methylmalonic acid when compared to the administration of free vitamin B_12_ [78]. Lecithin solid lipid microparticles (SLMPs) filled with vitamin B_12_ made by the spray cooling technique have been designed to increase the stability of the vitamin by Mazzocato et al. [79]. The prepared SLMPs had a spherical shape and the values of yield and EE ranged from 80.7 to 99.7% and from 76.7 to 101.1%, respectively. The formulations were stable for 120 days.

β-Glucan is known to significantly affect immunity [80], so it seems that of the above mentioned nanoformulations, the combination of vitamin B_12_ with β-glucan may have the greatest benefit in enhancing immunity [77].

### 2.3. Folic Acid

Folic acid (FA, Figure 2) is a water-soluble compound from the group of B vitamins. The active form of FA, tetrahydrofolate, is essential for the synthesis of nucleic acids (coenzyme of transferases), in hematopoiesis and especially important for normal growth and fetal development (helps cell division, promotes growth and tissue differentiation, especially of the nervous system) [67,81]. FA is found mainly in leafy vegetables, yeast and liver, but cooking destroys up to 95% of it [67]. Even with a high intake, it is not toxic, and excess is easily excreted in the urine. The absorption of FA ingested in foods is about 50%. Deficiency causes megaloblastic anemia; low intake increases the risk of depression; abortions and fetal damage occur in pregnant women [67,81].

FA encapsulated within conventional and emulsion-templated ALG-pectin hydrogels, proliposomes, and a combination thereof with sizes of 350 nm to 250 μm was characterized with good stability in a wide pH and temperature range in both dark and light conditions. At low pH, the release of FA was prevented, while in the intestine, FA was completely released via solubilization and carrier swelling. A 30–70% retention of FA was achieved at temperatures ≤90 °C when FA was encapsulated in emulsion-templated ALG-pectin beads and proliposomes, whereby the best protection was shown by proliposomes reinforced within a polysaccharide network. At pH 3.0 in the ALG-pectin beads, ≥70% of the recommended daily allowance of FA remained after 6 months of storage at room temperature and dark conditions [82]. FA-loaded ALG/CS nanolaminates fabricated by the layer-by-layer (LbL) technique showed enhanced stability under UV light as compared to free FA and a higher release rate of FA at pH 7 compared to pH 3, indicating their suitability as nutraceutical applications [83].

Electrospraying and nanospray were applied by Perez-Masia et al. [84] for entrapment of FA into spherical nano-, submicro-, and microcapsules using both a whey protein concentrate (WPC) matrix and a commercial resistant starch. While the electrospraying method provided smaller capsule sizes, greater EE was achieved using WPC, which could be connected with interactions between the protein and encapsulated FA. The enhanced thermal and UVA irradiation resistance of FA encapsulated in zein ultrafine fibers or nanocapsules fabricated by electrospinning and electrospraying was reported by do Evangelho [85]. At exposure of FA encapsulated in fibers to 180 °C, only 8.74% of FA was lost compared to 70% loss observed with free FA. Similarly, a 24 h exposure to UVA irradiation resulted in a 26% reduction in free FA, while only 10.86% FA was lost from FA encapsulated in nanocapsules. Consequently, it can be stated that zein ultrafine fibers or nanocapsules with encapsulated FA could be successfully applied in foods that require thermal processing or exposure to irradiation. Double emulsions fabricated by Assadpour et al. [86] with internal NEs consisting of a water-in-oil (W/O) system and with FA in the water phase, which were re-emulsified within an aqueous phase of pectin–WPC complexes achieved 82.3–95.0% EE of FA. The EE was affected predominantly by the interaction between WPC and dispersed phase content.

Nanocomplexes of 7S and 11S globulins isolated from defeated soy flour with FA were found to enhance the biomass formation of *Lactobacillus casei* BL23 in culture media, which was connected with the entry of the acid by the specific receptors concomitantly by the peptide receptors. It could be mentioned that the binding of FA resulted in protein aggregation via self-association. Such nanocomplexes can be used in nutraceutical and food industries [87]. The investigation of the binding of FA to β-lactoglobulin and type A gelatin and the formation of NPs/microparticles (MPs) in a pH range 3–7 showed that ionic bonds played a role in the binding of FA to proteins. At pH 3, 100% binding of FA to both tested proteins was achieved, and the increase in the molar FA/protein ratio resulted in a great increase in particle sizes. Both formulations protected FA at low pH occurring in the stomach and delivered it at the duodenum (pH 7) [88].

The investigation of FA encapsulation in different SiO_2_ porous supports showed that differently capped materials arrested the delivery of FA at low pH (corresponding to that of the stomach) but were able to deliver pronounced amounts of FA at neutral pH (as in the intestine). The application of SiO_2_ materials with a large pore entrance resulted in an initial fast release, while with the use of MCM-41, a sustained release was observed [89]. By functionalization of MCM-41 support with 3-[2-(2-aminoethylamino)ethylamino]propyltrimethoxysilane, gated polyamine-functionalized mesoporous SiO_2_ were prepared, which hindered the release of FA in gastric fluids (pH 2) and increasingly delivered FA in the presence of a simulated intestinal juice (pH 7.5) [90]. Amine-functionalized mesoporous SiO_2_ support loaded with FA incorporated in yogurt exhibited controlled FA release in intestine in contrast to acidic conditions, where the release was hindered and did not affect the physicochemical properties of yoghurt and lactic acid bacteria survival [91]. Using FA-loaded gated mesoporous SiO_2_ particles, the stability and controlled delivery of FA in fruit juices after consumption could be achieved due to modified FA bioavailability. Moreover, over-fortification risk can be reduced [92].

By intercalation of FA in the layered double hydroxides (LDHs), MgAl-LDH and ZnAl-LDH, nanostructured hybrids were formed, which, when formulated as single powders or as tablets, showed enhanced FA release at low pH compared to crystalline FA [93].

The above mentioned supplementation of yoghurt or fruit juice with FA-loaded functionalized mesoporous SiO_2_ opens up new opportunities for the development of new functional dairy products and refreshing beverages [91,92] and can be considered as formulations providing a significant benefit with added value from the aspect of immunity promotion along with the reduction of over-fortification risk.

### 2.4. Vitamin D

Vitamin D (calciferols, Figure 2) is the name for the steroid hormonal precursors of calcitriol, a hormone that affects the resorption of calcium and phosphate from the intestine, regulating the levels of calcium and phosphorus in the blood, so it is important for strong and undamaged bones [67,74,94]. It is important for the proper functioning of the immune system (long-term deficiency is associated with respiratory infections and influenza). It is important for alleviating immunodermatological problems [20,21,67]. Vitamin D affects approx. 200 different chemical reactions in the body and is found in all types of human cells and in all human tissues. Structurally, vitamin D occurs in two modifications: vitamin D_2_ (ergocalciferol) and vitamin D_3_ (cholecalciferol) [67]. Vitamin D_3_ is produced in the skin by the action of sunlight (UVB) from provitamin 7-dehydrocholesterol. This synthesis covers 80% of the daily requirement. The amount of vitamin D produced is reduced by protective creams, frequent baths in hot water, dry skin of the elderly, large amounts of melanin in the skin, body envelopes, and air pollution. In food, vitamin D_3_ is found in fish oil, liver, egg yolk, and milk. The recommended dose for adults is 2000–4000 IU per day with blood levels of 30–60 ng/mL. At least two-thirds of all people living in northern latitudes are deficient in vitamin D. In plants, the precursor of vitamin D_2_ is ergosterol or morphine [67,74,94].

Maurya et al. [95] comprehensively overviewed present findings related to fortification of food products with vitamin D with emphasis on factors affecting its bioavailability and the application of various suitable microencapsulation techniques, including liposomes, SLNPs, NLCs, NEs, spray drying, etc., which can be used for this purpose. Vitamin D NE-based delivery systems fabricated by spontaneous emulsification, which could be used in food industry, were reported by Guttoff et al. [96]. The NE-based delivery system was found to increase the in vitro bioavailability of vitamin D_3_ 3.94-fold, and according to the in vivo test, in which vitamin D_3_ NE and vitamin D_3_ coarse emulsion were used, the application of the nanoformulation resulted in a ca. two-fold increase in 25-hydroxycholecalciferol in serum compared to the coarse emulsion (an increase of 73% vs. 36%) [97].

Vitamin D_3_ incorporated into the polymeric complex of carboxymethyl chitosan (CMCS) with soy protein isolate (SPI) showing sizes of 162–243 nm, zeta potentials ranging from −10 to −20 mV, and EE up to 96.8% exhibited a ca. two-fold lower release in SGF (42.3% vs. 86.1%) but a ca. 4.4-fold higher release in SIF (36.0% vs. 8.2%) compared to NPs fabricated using SPI [98]. Spherical *N*,*N*-dimethylhexadecyl CMCS core-shell micelles with a positive charge (+50.7 mV) encapsulated vitamin D_3_ with 53.2% EE resulting in its improved solubility. These core-shell micelles released vitamin D_3_ at first rapidly; later its sustained release was observed [99]. Nanocomplexes prepared from ovalbumin, high-methoxylated pectin and encapsulated vitamin D_3_ showing high EE of 96.37% were characterized with electrostatic interactions, hydrogen bonding, and hydrophobic interactions among the three constituents and released only a small amount of vitamin D_3_ in SGF, while a large amount in SIF suggesting their potential to be used in food applications [100]. High amylose starch nanocarriers with particle sizes of 14.2–31.8 nm and a negative surface charge loaded with vitamin D_3_ and achieving 37.06–78.11% EE that were investigated for food fortification using milk as a model food supplementing Ca improved the bioavailability of vitamin D_3_ and masked the after taste, suggesting their potential to be used for the fortification of food with vitamin D_3_ [101]. In oil-in-water (O/W) Pickering emulsions stabilized by nanofibrillated cellulose (NFC; diameter: ca. 60 nm, length: several micrometers) encapsulating vitamin D_3_ containing 0.01% *w*/*w* vitamin D_3_, 9.99% *w*/*w* soybean oil, 0.10–0.70% *w*/*w* NFC as emulsifier at phosphate buffer of pH 7, the extent of lipid digestion and vitamin bioavailability decreased with increasing NFC concentration [102]. Mitbumrung et al. [103] encapsulated vitamin D_3_ in 10% wt soybean O/W Pickering emulsions stabilized by NFC or whey protein isolate (WPI) providing good stability to the emulsions via a combination of steric and electrostatic repulsion, where emulsions properties and EE were not affected by heating or ionic strength, and at highly acidic conditions (pH 2), particle size increased and EE showed a decrease. By an increase in NFC or WPI concentration, the stability and EE of the emulsions was improved and the encapsulated vitamin was effectively protected against environmental stresses occurring in industrial food production (e.g., pH changes, salt addition, and thermal processing).

The application of digestible oil (DO), indigestible oil (IO), or their mixture affected both the lipid digestion rate and the bioavailability of vitamin D_3_ encapsulated in NEs. The highest lipid digestion rate and vitamin bioavailability were observed with NEs using DO, the lowest one with those using IO, while comparable results were obtained with oil mixture (OM) consisting of 1:1 DO:IO mixed before homogenization and a 1:1 mixture consisting of DO and IO NEs mixed after homogenization. The maximum amount of vitamin D_3_ was estimated after ca. 30 min, and then its level showed a decrease during the following 24 h, which could be connected with an initial solubilization of the vitamin within the mixed micelles and following precipitation during prolonged incubation [104]. From O/W NEs prepared using various oils and natural surfactant, quillaja saponin, encapsulating vitamin D_3_, the release of free fatty acids during lipid digestion in a simulated gastrointestinal tract (GIT) model decreased as follows: medium chain triglycerides (MCT) > corn oil ≈ fish oil > orange oil > mineral oil, while the bioavailability of vitamin D_3_ increased in following order MCT < mineral oil< orange oil < fish oil ≈ corn oil suggesting that the greatest increase in vitamin D_3_ bioavailability can be obtained with NEs fabricated with long chain triglycerides (corn or fish oil) [105]. By blending caprylic-/capric triglyceride and Kolliphor HS^®^15, vitamin D_3_ and sodium chloride in optimal ratio, Maurya and Aggarwal [106] prepared a formulation with encapsulated vitamin able to tolerate environmental stress conditions, and based on sensory evaluation it was found to be suitable for fortification of vitamin D_3_ in “Lassi”, a milk based beverage. Uncoated nanoliposomes loaded with vitamins D_3_ and K_2_ were fabricated using a novel, a semi continuous technique based on simil-microfluidic principles and covered with CS to enhance the mucoadhesiveness and the stability of the liposomal structures, whereby CS was tested as covering material. Such polymer–lipid hybrid NPs encapsulating the above-mentioned vitamins were characterized with improved stability, loading, and mucoadhesiveness, suggesting their potential to be used in nutraceutical applications [107].

Vitamin D_3_ was incorporated into an NLC consisting of Precirol^®^ (glyceryl palmitostearate) as a solid lipid and octyl octanoate as a liquid lipid. The surface of these NLCs was coated with either Poloxamer 407 or Tween 20. Both of these surfactants prevented agglomeration during the homogenization process while increasing intestinal absorption of the entire formulation, suggesting that NLCs can be used as an excellent carrier to enrich beverages with vitamin D_3_ [108].

Berino et al. [109] studied the interaction of vitamin D_3_ with β-lactoglobulin at high vitamin/protein ratios and found that when 100 μM vitamin D_3_ and 20 μM β-lactoglobulin in 20 mM phosphate buffer at pH 7.0 were used, vitamin D_3_ interacted in the hydrophobic calix in the protein, and the binding of the vitamin caused conformational changes in the secondary β-lactoglobulin structure. With the increasing vitamin concentration, the proportion of bound vitamin increased likely due to a cooperative phenomenon and/or a stacking process. Moeller et al. [110] enriched low fat yoghurt by spray- and freeze-dried casein micelles loaded with vitamin D_2_ maintaining constant vitamin content in powders during 4 months of storage. Based on the results of an in vitro proteolysis, when 90% of the vitamin D_2_ encapsulated in dry casein micelles remained active compared to 67% of free vitamin D_2_, it was assumed that after proteolysis, the vitamin will be ultimately available in the lumen. Using the optimal loading of vitamin D_3_ into re-assembled casein micelles (1.38–1.46 mg/100 mg casein) performed at 4.9 mM PO_4_^3−^, 4.0 mM citrate, and 26.1 mM Ca, more vitamin D_3_ was retained in the re-assembled casein micelles than in control powders during storage, however its loss after 21 days of refrigerated storage with light exposure was comparable with that of the control fortified milks suggesting that re-assembled casein micelles can improve vitamin D_3_ stability during dry storage [111]. The highly protective effect of the re-assembled casein micelles against gastric degradation of vitamin D_3_ resulted in its four-fold higher bioavailability compared to the free vitamin D_3_ [112].

Vitamin D_3_ and potato protein co-assemblies formed in phosphate buffer at pH 2.5 provided transparent solutions that were able to significantly protect and reduce vitamin D_3_ losses during pasteurization. Testing performed under different storage conditions suggests that potato protein could be used as a good carrier of vitamin D_3_ and the entire stable formulation could be used to fortify clear beverages, other foods, and drink products with vitamin D_3_ [113].

Pea protein-stabilized NEs with particle sizes of 170–350 nm and zeta-potential of −25 mV, which were characterized with good stability and the high EE of D vitamin (94–96%) exhibited considerably higher cellular uptake than emulsions fabricated using a combination of protein and lecithin, the cellular uptake of NEs with particle sizes of 233 nm being higher than that observed with NEs of 350 nm. Evently the transport efficiency of vitamin D in NEs with smaller particle sizes across Caco-2 cell was 5.3-fold greater than that of free vitamin D suspension, suggesting that pea protein could be considered as an effective emulsifier for fabrication of food NEs ensuring the improved bioavailability of vitamin D [114]. Pea protein isolate (PPI), the function properties of which were modified using pH-shifting and sonication combined treatment, was applied to prepare NEs encapsulating vitamin D_3_. The NEs ensured good protection of vitamin D_3_ against UV radiation, were stable during 30-day storage, and showed ameliorated antioxidant activity as well as markedly higher recovery of vitamin D_3_ in micelles through in vitro digestion, suggesting that such NEs could be used for protection and delivery of nutraceuticals in foods [115]. The application of vitamin D_3_ encapsulated in PPI NE at the dose of 81 μg daily to vitamin D deficient rats for one week resulted in higher serum 25-hydroxycholecalciferol levels compared to the control as well as in changes in serum parathyroid hormone, Ca, P, and *alkaline phosphatase* levels as compared to the controls. Hence, vitamin D_3_ encapsulated in PPI-based NEs improved its absorption and restored its status and biomarkers of bone resorption in vitamin D deficient rats [116].

Salvia-Trujillo et al. [117] investigated the impact of the initial lipid droplet size on the in vitro bioavailability and in vivo absorption of vitamin D_2_ encapsulated in O/W NE. The in vitro studies, in which vitamin D_2_-loaded lipid droplets were passed through a simulated GIT, showed the highest bioavailability of the vitamin with the emulsions containing the smallest droplets, because they were digested more rapidly than larger ones and were able to form quickly mixed micelles in the small intestine capable to solubilize the lipophilic vitamin. On the other hand, in the in vivo rat feeding studies, the highest absorption of vitamin D_2_ was observed with NEs containing the largest droplets. This discrepancy could be connected with the fact that the simulated GIT cannot precisely reflect the complexity of a real GIT and by the applied in vivo approach, the changes in vitamin levels in the blood were not monitored over time.

Using mixed surfactant (Tween 80 and soya lecithin), vitamin D NEs were fabricated by ultrasonic homogenization showing droplet sizes of 140.15 nm and 155.5 nm after 2 months storage at 4 and 25 °C, respectively; after 30 days of storage at 4 and 25 °C, the NEs retained 74.4 ± 1.2 and 55.3 ± 2.1% of vitamin D, suggesting their suitability to be used in food and beverages [118]. The optimized vitamin D NEs fabricated by Mehmood et al. [119] using ultrasonication and lecithin and Tween 80 at a ratio 2:3 showed the size of 112.36 ± 3.6 nm and the vitamin D retention of 76.65 ± 1.7%. The higher release of vitamin D_3_ under simulated intestinal condition was observed from NEs co-encapsulating vitamin D_3_ and saffron petals’ bioactive compounds, which were stabilized with basil seed gum and prepared using high pressure and ultrasound compared to those fabricated using WPC and Tween 80 emulsifiers [120]. The investigation of a series of 2 wt% O/W emulsions containing different initial levels and locations of CS NPs and Tween 80 with encapsulated vitamin D_3_ showed that the NEs stabilized with Tween 8 exhibited 30% higher lipid digestion and 45% higher vitamin D_3_ bioavailability than those prepared with CS NPs, and the resulting effect depended on the applied ratio of CS NPs and Tween 80. It can be assumed that a layer of CS NPs limit the lipase to reach the lipid phase, the significant aggregation of droplets coated with CS NPs reduced the area of lipids, which is accessible to the lipase, and the positively charged CS NPs bound to anionic bile acids, fatty acids, or lipase. While the slowing of lipid digestion by CS NPs would be favorable at application in high-satiety foods, the reduced bioavailability of vitamin D is unfavorable [121]. O/W NEs prepared using Tween 20, soybean lecithin, and their mixtures as emulsifiers and soybean oil or mixtures of the oil with cocoa butter as a dispersed oil phase using high pressure homogenization, showing oil droplets encapsulating vitamin D_3_ with average diameters <200 nm, maintained physical stability for several weeks. In systems stabilized by Tweens, partial vitamin’s embedment in the interface of NEs was observed. The whole-fat milk fortified with vitamin D_3_ enriched NEs remained stable to particle aggregation and gravitational separation for at least 10 days [122].

Leaving aside the above mentioned combined nanoformulation of vitamin D with vitamin C [72], it appears that the described nanoencapsulation of vitamin D into casein [123], micelles and their application to yoghurt [110] has the greatest benefit for immunity of the nanoformulations discussed above.

### 2.5. Vitamin E

Vitamin E (tocopherols, Figure 2) is a derivative of 6-hydroxychroman and a hydrophobic phytyl chain, which causes water insolubility and good fat solubility. The most common is d-α-tocopherol (α-Toc) with the strongest antioxidant activity, which is contained in wheat germ oil, sunflower seeds, almonds, hazelnuts, butter, milk, peanuts, soybeans, lettuce and mammalian meat. Vitamin E is destroyed during cooking and food processing, including freezing [67]. The need for vitamin E increases with the increased intake of unsaturated fats. In the body, it is part of the membranes, where it acts in the first line of defense against peroxidation of polyenoic acids of biological membranes. Tocopherol stops radical chain reactions and formed tocopherol radical can subsequently react with vitamin C, reduced glutathione, coenzyme Q10, or with itself [67,124]. In addition to the antioxidant action of tocopherols, it stabilizes membranes, affects membrane permeability and acts as an inhibitor of protein kinase C. As the most important antioxidant in the body, Vitamin E helps to slow aging and has been shown to prevent cancer [67,125]. Vitamin E deficiency is associated with disorders of fat absorption or distribution or after bowel resection and can cause neurological problems, decreased immunity, or infertility. Newborns develop anemia. The prolonged overuse of high doses worsens the absorption of vitamin K [67,124,125].

The spray freeze-drying based microencapsulation technique applied for the encapsulation of α-Toc using whey protein (WP) enhanced its oral bioavailability and can be successfully applied also for increasing the bioavailability of other poorly water-soluble bioactive compounds [126]. Jaberi et al. [127] published optimized formulation parameters related to the fabrication of α-Toc nanodispersions using the low-energy solvent displacement technique; these nanodispersions can be applied in several water-based foods. α-Toc-loaded nanocapsules fabricated using octenyl succinic anhydride-modified starches of low molecular weight with the degree of substitution 5.1 and 2.7%, serving as emulsifiers and wall materials, were able to retain ca. 50% of α-Toc after 60 days of storage at 4–35 °C; these stable nanocapsules with encapsulated α-Toc could be used in beverage applications [128].

CS/tripolyphosphate-nanoliposomes core-shell nanocomplexes used as carriers for encapsulation of α-Toc retained >80% of α-Toc during one month storage; the coated liposomes were characterized with increased stability against temperature and retained 92% and 97% of α-Toc after exposure to 65 °C for 30 min and 80 °C for 16 s, respectively [129]. In milk phospholipid assemblies encapsulating vitamin C and α-Toc, the polar parts of phospholipids formed H-bonds with OH groups of vitamin C, and following incorporation of C or E vitamin into phospholipid assemblies, the chemical conformation of the complexes was modified. Phospholipid—vitamin C phytosomes showed superb biocompatibility on intestinal epithelial cells achieving the cellular uptake of vitamin C of 29.06 ± 1.18% exceeding that observed for liposomes (24.14 ± 0.60%) and for vitamin C aqueous solution (1.17 ± 0.70%) [130].

Nanoemulsified vitamin E markedly enhanced total serum levels of α-Toc in rats, and no symptoms of acute toxicity after the administration of 2000 mg/kg of nanoscale α-Toc were observed 14 days from the administration [131]. Vitamin E encapsulated in NEs fabricated by the emulsion phase inversion method using oil short-, medium- and long-chains trigycerides as carrier were found to be physically stable to short-term heat shock (30–90 °C, 30 min), pH range of 2.0–8.5, salt, and long term storage (60 days), although during heat processing and long-term storage of samples, pronounced degradation of α-Toc was observed. The NEs showed higher stability at 4 °C than at 40 °C, and in samples stored in dark, enhanced α-Toc retention was observed compared to those stored in the light. The susceptibility to higher temperature (>25 °C) was estimated in NEs fabricated with short-chain trigycerides [132]. α-Toc formulated with coconut oil gave an NE with the encapsulation capacity of 9.5 mg α-Toc per mL of oil, and almost 100% release of the loaded active ingredient was observed within 24 h, the release being predominantly kinetically controlled. The mentioned formulation proved to be sufficiently stable and biocompatible [133].

The core-shell nanoencapsulation of α-TOC was achieved by blending sodium oleate (NaOl) and rebaudioside A (RebA), and H-bonds and hydrophobic interactions were estimated as the major forces in α-Toc-NaOl/RebA complexes. Nanoencapsulation considerably enhanced the antioxidant activity of α-Toc, and ca. 67.9% of α-Toc was released from the formulation at 25 °C after 90 h [134]. α-TOC NEs with the mean particle size of 277 nm prepared using sunflower oil, saponin, and water showed three-fold higher bioavailability in male Wistar rats compared with conventional emulsion with 4.64-fold greater droplet diameters, and in both emulsion types, droplet flocculation and coalescence during in vivo digestion was observed. During thermal processing (30–90 °C), mechanical stress, and long-term storage, the saponin-coated NE was stable to droplet coalescence [135]. The encapsulation of vitamin E in O/W NE using corn oil as a carrier oil and quillaja saponin as a biosurfactant was performed by Lv et al. [136]. A rise of α-Toc in NEs reduced the storage stability of the NEs, bioavailability, as well as the rate and extent of lipid hydrolysis in the small intestine, likely due to the inhibition of the lipase ability to reach the triacylglycerols inside the lipid droplets. With optimized NE, 53.9% bioavailability of vitamin E was observed. NE-based vitamin E delivery systems prepared using quillaja saponin and lecithin, i.e., surfactants of natural origin, achieved the smallest droplet diameter of *d*_32_ = 130 nm for lecithin and 120 nm for quillaja saponin at the ratio vitamin E to orange oil of 50%:50%. At pH 2, the NEs formed using both surfactants were unstable, because a reduction in droplet charge accompanied with a loss of electrostatic repulsion and NE instability was observed also in the presence of >100 mM NaCl for lecithin and ≥400 mM NaCl for quillaja saponin. On the other hand, the NE maintained stability in a wide temperature range (30–90 °C) at pH 7 [137]. Fang et al. [138] co-encapsulated α-Toc and resveratrol (RES)/naringenin in emulsions stabilized by WPI. The total encapsulation of α-Toc was achieved with the 3.3-fold higher amount of α-Toc within oil droplets compared to that bound by free WPI. The total EE of RES and naringenin was lower (52% and 58%, respectively). The digestive stability of α-Toc was improved only at co-encapsulation with RES. On the other hand, the presence of α-Toc in the emulsion did not affect the digestive stability of co-encapsulated RES/naringenin.

Schroder et al. [139] investigated the effect of emulsifier type and lipid composition on particle morphology and antioxidant stability of colloidal lipid particles (CLPs) encapsulating α-Toc and found that Tween emulsifiers supported tripalmitin crystallization into highly ordered lath-like particles, while at the application of sodium caseinate emulsifier, less ordered spherical particles were formed. α-Toc encapsulated in tripalmitin-based CLPs stabilized by Tween 40 showed the most rapid degradation, which may be attributed to its expulsion to the particle surface induced by lipid crystallization suggesting that in this case, the lipid crystallization did not protect sufficiently entrapped vitamin E.

In NEs enriched with β-carotene (β-Car) and α-Toc prepared using MCT as carrier oils and octenyl succinic anhydride modified starch or Tween-80 as emulsifiers, the α-Toc acts as an antioxidant protected β-Car from degradation, however this was not observed when flax seed oil was used as carrier oil. The improvement of the oxidative stability of NEs and the retention of α-Toc and β-Car in NEs were achieved by incorporation of eugenol. In α-Toc and β-Car-loaded NEs stabilized by octenyl succinic anhydride modified starch forming likely thicker protective layer around oil droplets than Tween 80, ca. 42% retention of β-Car and ca. 90% retention of α-Toc was estimated after 4 weeks of storage at 40 °C [140]. α-Toc acting as a potent antioxidant at 0.10% wt. showed the highest efficacy compared to *tert*-butylhydroquinone and ascorbyl palmitate in ensuring the thermal and light stability of encapsulated β-Car in dilute O/W emulsions prepared using GA [141]. Also the α-Toc based O/W NE stabilized with sodium stearoyl lactate surfactant prepared by ultrasonic emulsification, which was stable up to 90 days in salt solution (50–200 mM) and different pH conditions, was applied as a delivery vehicle for antioxidants, curcumin (CUR) and benzylisothiocyanate (BITC). The average size of free NE was 38.68 nm, the average sizes of CUR-loaded, BITC-loaded and CUR+BITC-loaded NEs were 49.67 nm, 5.83, and 53.52 nm, respectively. The antioxidant effectiveness of α-Toc based NE (IC_50_ of 85.46 μM) was synergistically enhanced with the encapsulation of CUR and BITC [142].

As WP is widely used by food manufacturers for its nutritional and functional value and health-promotion effects [143], including immunomodulation, antihypertension, and cardioprotection [144]. The antioxidant properties of WP have been used to produce a natural antioxidant used as a food additive [145]. Therefore, in the shadow of these facts, the combination of vitamin E with WP [126] can be considered the most beneficial for human health of the above nanoformulations.

## 3. Minerals

Trace elements (<1 g) from the group of metals include Fe, Zn, Cu, Mn, Mo, Cr, Co, V, and Sn, and from the group of non-metals, they are F, I, Se, Si, and B. This part will focus only on iron, selenium, and zinc [67].

### 3.1. Iron

Iron is needed for the production of red blood cells and a number of vital functions (growth, reproduction, wound healing, and immunity). Hemoglobin contains about 65% Fe, myoglobin in the muscles 10%, and the rest of the iron is present in the liver, kidneys, bone marrow and other organs. In a healthy person, there is a daily loss of iron in the amount of approximately 1 mg; in women during menstruation or breastfeeding, this value can be approximately double, but also higher. The absorption of iron from food depends on the source. The absorption from plant materials is low (approx. 5%), while from meat, it is the highest (up to 24%). The absorption of iron is negatively affected by polyphenols, tannins, calcium, and vegetable proteins. The bioavailability of iron is improved by vitamin C, some organic acids, and meat; vitamin A deficiency impairs its utilization. Iron deficiency is manifested by anemia [67,146].

The mechanism of fat-binding and fat-contenting of Fe_3_O_4_ (FeO.Fe_2_O_3_) NPs applied as a food supplement by the “two-layer coordination” model using NPs based on HCO_3_^−^ and Fe_3_O_4_, coated with linoleic acid and unrefined sunflower oil was discussed by Tsykhanovska et al. [147]. Kruhlova et al. [148] reported that the nutritional supplement “Magnetofood” based on NPs of two and three valence iron oxides can be introduced to recipes of bakery, flour confectionery, meat, pasty-marmalade products, cheesecakes, and whipped desserts, because it can support resource conservation during the production of food products, which subsequently exhibit high organoleptic, functional, and technological properties and extended shelf-life. This would seem to be a suitable supplement for anemic patients. The potential of nanostructured oxides and phosphates of Fe and atomically mixed Fe/Zn in nutritional applications was discussed by Zimmermann and Hilty [149].

### 3.2. Selenium

Although selenium is an essential trace element (Se content in the human body is in the range of 13–20 mg, the daily dose of Se must not exceed 200 mg/day), it is highly toxic at a dose >800 mg/day [67]. Elemental Se and selenides have low bioavailability, and selenides and selenites are very toxic. Se deficiency (<60% normal) may occur in patients with intestinal dysfunction, in patients with complete parenteral nutrition, and in patients over 90 years of age. Se acts as a cofactor for antioxidant enzymes (glutathione peroxidase), so it was hypothesized that it may reduce the risk of cancer, but research has not shown support for these claims [150,151]. However, it was found that decreased Se levels can adversely affect the cardiovascular system (increased risk of myocardial infarction and vascular disease). A lack of Se in the diet of pregnant women can adversely affect fetal development, because the thyroid gland and every cell that uses thyroid hormone uses Se [67,150,151].

The investigation of the impact of SeNPs on a diverse and mature broiler caecal microbiota showed that they can be used in poultry production for targeted *Enterococcus cecorum* control without significant disturbance to the total microbial community [152]. Mates et al. [153] focused attention on fabrication mode, the structure, and morphology of SeNPs and their possible use in biomedicine and food technology.

Some lactic acid bacteria can biotransform toxic SeO_2_^2−^ into SeNPs and Se-amino acids. *Lactobacillus brevis* CRL 2051 and *Fructobacillus tropaeoli* CRL 2034 in de Man-Rogosa-Sharpe (MRS) culture medium with and without SeO_2_^2−^ were used for the inoculation of fermented fruit juice-milk (FJM) beverage. The selenization did not affect the growth of lactic acid bacteria in the FJM drink; the highest Se concentration was observed in the fermented beverage with selenized *L. brevis*. A decrease in the cell count of selenized cells of *L. brevis* by 1.1 log_10_(CFU/mL) was observed under storage. On the other hand, cell viability was not affected for non-selenized *L. brevis* or both selenized and control cells of *F. tropaeoli*, and an increase in the resistance of *F. tropaeoli* selenized cells during digestion of the fermented FJM by 1 log_10_(CFU/mL) was estimated [154].

SeNPs decorated with CS (510 kDa) were stable for >45 days and showed higher antioxidant activities than the undecorated SeNPs suggesting their suitability to be used in functional foods as additives [155]. SeNPs of 60 nm encapsulated in CS microspheres applied to Wistar rats achieved a ca. eight-fold higher LC_50_ value compared to selenite and increased Se retention in Se-deficient animals. Moreover, they considerably suppressed the ethanol-induced gastric mucosal damage, which could be connected with their superb antioxidant activity able to reduce the levels of NO, suggesting that this nanoformulation is suitable for oral delivery of SeNPs as a Se-supplement [156]. SeNPs-loaded CS/chitooligosaccharide (COS) MPs fabricated by spray-drying of a mixture of SeNPs, CS, and COS and characterized by smooth or wrinkled surface, hollow core, and COS body filled with SeNPs–CS nanobeads were found to protect mice from ethanol-induced oxidative stress due to their strong antioxidant activity. Reduced lipid and protein oxidation was connected with the enhanced activities of glutathione peroxidase, superoxide dismutase, and catalase and effective scavenging of reactive oxygen species (ROS) [157].

Spherical pectin-stabilized SeNPs prepared at a ratio Se/pectin of 1:2, showing average particle size of 41 nm, remained stable in acidic solutions for at least 1 month and exhibited strong 2,2-diphenyl-1-picrylhydrazyl (DPPH) radical scavenging ability and antioxidant capacity [158]. Pectin-stabilized SeNPs encapsulating CUR with the particle size of 119 nm and the EE of ca. 60.6% showed pH-dependent and controlled CUR release in vitro. CUR solubility in these NPs showed a ca. 500-fold increase as compared with free CUR, and their free radical scavenging ability and antioxidant capacity exceeded those of pectin-stabilized SeNPs [159].

In SeNPs fabricated using tilapia polypeptides as a stabilizing agent, the Se^0^ reduced from Na_2_SeO_3_ aggregated to a SeNPs core and was subsequently encapsulated by the tilapia polypeptides. A solution of such SeNPs was found to be relatively stable in neutral and alkaline environment with best storage stability at pH 8 [160].

Since Se is generally perceived as an element strengthening immunity and reducing oxidative stress, the supplementation of food products with Se nanoformulations can play an important role. Therefore, it would be mostly desirable to add nanoselen in yogurt with probiotic microorganisms [154].

### 3.3. Zinc

Zinc is required for the activity of more than 200 Zn-dependent metalloenzymes (e.g., carbonic anhydrase, alcohol dehydrogenase, lactate dehydrogenase, alkaline phosphatase, superoxide dismutase, etc.), necessary for DNA synthesis and for the function of some DNA-binding proteins. Zn is necessary for cell proliferation, immune responses, and the stabilization of the hormone–receptor complex [67,161]. Zn sources are meat and other foods rich in protein, whole grains, legumes, root vegetables. Unlike Cu and Fe, Zn is not stored in the liver [67]. Zn deficiency may be genetically determined or acquired primarily (i.e., by an inappropriate diet) or secondarily (i.e., by a disease leading to insufficient absorption, e.g., acrodermatis enteropathica—Danbolt’s disease). Zn deficiency is one of the most common micronutrient deficiencies in the world and can cause dermatitis, alopecia, poor wound healing, severe diarrhea, neuropsychiatric disorders, hypogonadism, or low immunity. In pregnant women, severe zinc deficiency is associated with birth defects and miscarriages [162].

The addition of ZnO NPs in food matrices can affect biological systems due to interactions between NPs and food components. It was found that saccharides considerably affected the hydrodynamic radii and zeta potentials of ZnO NPs. The evaluation of biological responses of ZnO NPs dispersed in different saccharides in human intestinal cells and rats showed that NPs in all tested saccharides (acacia honey, sugar mixtures containing equivalent amounts of fructose, glucose, sucrose, and maltose, and monosaccharide solutions) increased the inhibition of cell proliferation and enhanced cellular uptake. A great enhancement of oral absorption of ZnO NPs was achieved with 5% glucose, which corresponded to the results observed with intestinal transport results [163]. The hydrodynamic radii and zeta potentials of bulk ZnO and ZnO NPs in biofluids were found to change in different ways. The size of ZnO NPs did not affect ZnO solubility and its intestinal transport mechanism, and albumin, fibrinogen, and fibronectin played a role in particle-plasma protein corona, whereby ZnO NPs interacted more strongly with plasma proteins [164].

Biocompatible xanthan gum capped ZnO microstars showing both hexagonal phase and starlike structures with the average particle size of 358 nm, which can be used for the fortification of food with Zn, were developed by Ebrahiminezhad et al. [165]. These microstars were biocompatible and did not exhibit antimicrobial effects against *Escherichia coli*, *Bacillus licheniformis*, *Bacillus subtilis*, and *Bacillus sphaericus*, suggesting their suitability to be used for dietary supplementation and food fortification. In a review paper, Swain et al. [166] described the beneficial effects of ZnO NPs and their possible use as an alternative to conventional Zn mineral supplements for different categories of human and livestock. The quantitative polymerase chain reaction (PCR) test showed that the combined administration of ZnO NPs and the fermentation liquor of *Lactobacillus plantarum* BLPL03 (FLL) isolated from GIT of healthy postweaning piglets synergistically elevated the faecal number of *Bifidobacterium* 73–19-fold, while reducing potential enteropathogenic bacteria *Enterobacteriaceae* and *Clostridium perfringen*s in mice challenged with *Salmonlla typhimurium*. The co-application of 20 mg/kg of ZnO NPs and FLL resulted in enhanced final body weight and reduced feed conversion ratio and diarrhoea incidence in weaned piglets, and the administration of a mixture of ZnO NPs and FLL resulted in a dramatic rise of the faecal *Bifidobacterium* and *Lactobacillus* of piglets. The administration of WP NPs with incorporated zinc citrate with sizes of 142, 196, and 228 nm and zeta potentials −114, −85, and −79 mV to CCl_4_-treated albino rats counteracted the disturbances in biochemical parameters, gene expression, and histological changes as well as adverse effects of oxidative stress. Thus, WP NPs coating of Zn citrate in food supplements can enhance its effect and counteract the side effect of excess Zn [167].

On the other hand, daily exposure to inorganic NPs through various foodstuffs can evoke immune dysfunctions in the gut connected with favored colonization of the intestine by pathobionts at the expense of beneficial bacterial strains. Therefore, the impact of such NPs on the gut microbiome should be considered in human health risk assessment, and increased attention should be devoted to NPs showing antimicrobial activities [168]. Senapati et al. [169] investigated the immunotoxic potential of ZnO NPs after sub-acute exposure in different ages of BALB/c mice. While in aged mice, pronounced changes in CD4- and CD8-cells, levels of interleukin-6 (IL-6), interferon gamma (IFNγ), and tumor necrosis factor alpha (TNFα) as well as a considerable increase in the expression levels of mitogen activated protein kinase (MAPK) cascade proteins (phospho-ERK1/2, phospho-JNK and phospho-p38) and ROS were observed, in juvenile mice an increase in ROS, IL-6, and TNFα levels was estimated, but in adult mice, no significant changes were observed. Consequently, it is evident that the aged mice are more sensitive to ZnO NP induced immunotoxicity.

As mentioned above, WP has many benefits for human health [170]. Therefore, as in the case of nanoformulation of vitamin E with WP [126], it seems that the stabilization of nanoformulation of Zn citrate by WP [167] is especially advantageous for human health.

## 4. Antioxidants

### 4.1. Carotenoids

**Figure 3:**
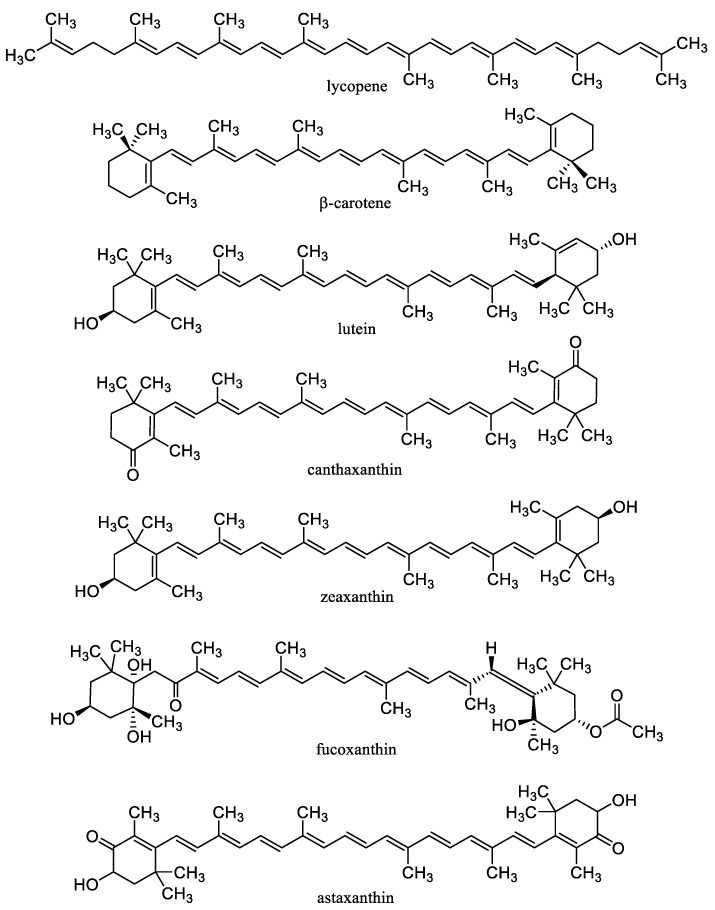
Structures of carotenoids affecting immunity.

Carotenoids (Figure 3) including carotenes xanthophylls are lipophilic tetraterpenoid dyes of plants, fungi, algae, microorganisms, and animals showing considerable antioxidant effects and can prevent damage to cells and DNA by the sun’s rays. Carotenes include lycopene, from which either α-carotene, which is further converted to lutein, or β-carotene (β-Car) is formed. Carotenoids include, for example, astaxanthin, zeaxanthin, and retinoids (vitamin A and its derivatives) [67,171]. β-Car is the most important provitamin of vitamin A. It is also an important antioxidant, and its deficiency increases the risk of cancer and reduces the body’s overall defenses. If vitamin A is not supplied to the body, significant health risks associated with vitamin A deficiency can occur [67]. β-Car plays an important role in protecting the skin from sun damage. There is no health problem with acute β-Car overdose (unlike vitamin A). The *cis*-form of β-Car formed increasingly at heat treatment is less absorbed, but it is a stronger antioxidant and remains longer in tissues. On the other hand, the all-*trans* form occurring in synthetic β-Car is more active as provitamin A [171,172]. β-Car is a food additive E160a and is used as colorant in cheese, margarine, ice cream, yogurt, mayonnaise, lemonade, puddings, or confectionery [67].

Composite phospholipid–CS vesicles (chitosomes) fabricated by combining the liposomal preparation and the layer self-assembly deposition technique, which could serve as delivery systems for lycopene, β-Car, lutein, and canthaxanthin were designed by Tan et al. [173]. Electrostatic and hydrophobic interaction of CS resulted in restricted motion freedom of lipid molecules and their enhanced ordering at the polar head group region and the hydrophobic core of the membrane, which contributed to the stability of carotenoid-loaded liposomes under heating, GI stress, and centrifugal sedimentation. The protection of β-Car by the liposomal membrane was superior to that of lycopene and canthaxanthin. A comprehensive review focused on carotenoid-loaded nanocarriers, including nanoliposomes, nanoemulsions, biopolymeric nanocarriers (polysaccharides and proteins), and lipid-based nanocarriers, was presented by Rehman et al. [174]. Choi and McClements [175] outlined strategies for improving formulation, stability, and functionality of NEs used as delivery systems for lipophilic nutraceuticals, including carotenoids, which can markedly enhance the bioavailability of encapsulated compounds.

Lycopene-loaded SLNPs prepared using Precirol^®^ ATO5 by hot homogenization (125 ± 3.89 nm; zeta potential of −10.06 ± 0.08 mV) showing the EE of 98.4% were stable in aqueous medium over 2 months when kept at 4 °C [176]. The investigation of yogurt incorporating zeaxanthin (ZX) NPs and ZX after 28 days of storage showed higher ZX retention in yogurt with ZX NPs than in yogurt with ZX NEs (22.31 ± 2.53% and 16.84 ± 0.53%, respectively). The incorporation of ZX NPs did not change the sensory properties of yogurt, while nanoencapsulation provided a controlled release of ZX after in vitro digestion [177]. Optimized ultrasound-mediated fucoxanthin oil rich NEs stabilized by κ-carrageenan designed by Saravana et al. [178] improved oxidative stability, in vitro digestion, and bioavailability of fucoxanthin and have potential to be used as a delivery system for seaweed oil applications in functional foods and beverages.

NEs fabricated using long chain triglyceride oils (flaxseed, olive and corn oil) markedly increased the bioavailability of astaxanthin (AST) in a simulated GIT model due to the formation of mixed micelles able to solubilize the hydrophobic AST. Free fatty acid (FFA) unsaturation and chain length affected the lipid digestion and micelle formation, and the final amount of FFAs released and AST bioavailability decreased as follows: olive oil > flaxseed oil > corn oil [179]. NEs prepared using various emulsions such as WPI, polymerized WP (PWP), WPI–lecithin, PWP–lecithin, lecithin, and Tween 20 encapsulating AST showed droplet sizes of 194–287 nm and EE >90%, good physicochemical stability during storage at 4 °C, and a considerably higher uptake of AST by Caco-2 cells compared to free AST. The highest cellular uptake of AST (10.0 ± 0.2%) was observed using NEs prepared using WPI, while the lowest one was observed for NEs fabricated using Tween 20 (2.1 ± 0.1%) [180]. AST-enriched NEs fabricated using caseinate as a emulsifier with the droplet diameter of 230 nm and the zeta potential of −40 mV remained physically stable in temperature range of 5–70 °C, and the chemical stability of AST was only slightly affected by solution pH, ionic strength, and light exposure, except at pH 4 and 5; droplet aggregation was observed at pH values near the isoelectric point of caseinate [181]. Hence, caseinate-stabilized NEs could be recommended as delivery systems for AST in functional foods and beverages. It seems that the last two formulations—combinations of WP [180] or caseinate [181] with carotenoids—have the best effect for immunity.

#### β-Carotene

β-Car is a precursor to vitamin A (retinol), which is transformed into retinol by the liver, according to the body’s needs. β-Car encapsulated in NEs underwent chemical degradation when stored at elevated temperatures. On the other hand, the incorporation of β-Car-loaded lipid droplets into hydrogel beads formed using 0.5 and 1.0% ALG strongly improved its chemical stability. The rate and extent of lipid digestion at simulated GIT conditions decreased as follows: free lipid droplets (NE) > 0.5% ALG beads > 1% ALG beads; however, the bioavailability of encapsulated β-Car in free lipid droplets was higher than that in hydrogel beads, but the best protection against degradation was observed with 1% ALG beads [182]. β-Car NEs lipid droplets, which were stabilized by chlorogenic acid–lactoferrin–polydextrose conjugate were characterized with good droplet stability to droplet aggregation under simulated GIT conditions and improved β-Car bioavailability suggesting that such NEs could be used as carriers of hydrophobic nutraceuticals, providing effective protection from degradation [183]. Protein-type emulsifiers and additional antioxidants are effective in protecting β-Car encapsulated in emulsions from degradation. The bioavailability of β-Car from consumed food is higher when it is encapsulated in smaller oil droplets containing long-chain fatty acids. Recent progress in emulsion-based delivery systems for β-Car including multilayer emulsions, solid lipid particles, and Pickering emulsions was summarized by Mao et al. [184]. Native casein micelles separated from skimmed milk and loaded with β-Car at 2 °C and pH 5.5 provided total recovery rates of >79% after the back-extraction of β-Car, where 94% of β-Car was associated with the casein micelles [185]. Adding maltodextrin (MDX) to β-Car emulsions stabilized by sodium caseinate resulted in excellent stability over 3 months of storage at 4 °C (>92.1% retention of β-Car compared to 62.7% retention in emulsion without MDX) and superb freeze–thaw stability [186]. Catechin—egg white protein conjugates used as antioxidant emulsifiers at fabrication of β-Car emulsions provided them enhanced resistance against thermal processing and high ionic strengths due to stronger steric repulsion between the oil droplets, and their antioxidant and interfacial activities considerably contributed to a decrease in β-Car degradation rate during storage [187].

Excipient NEs formulated from long chain triglycerides strongly improved β-Car bioavailability from tablets (20%) and slightly improved it from soft gels (5%). On the other hand, β-Car bioavailability from NEs prepared using MCT was improved only slightly, which could be connected with the ability of β-Car to be incorporated into large mixed micelles formed by long chain triglyceride digestion but not by small ones formed by MCT digestion [188]. However, excipient emulsions showed a considerably lower impact on bioavailability of phenolics, i.e., smaller more polar molecules, which can be more easily solubilized in aqueous intestinal fluid [189].

The bioavailability of carotenoids from tomatoes using emulsions with the droplet sizes (*d*_32_) of 150 nm, 400 nm, and 22.3 μm decreased with increasing initial droplet size due to the more efficient extraction of carotenoids from tomato tissue by smaller droplets, which were also digested faster, which resulted in more rapid mixed micelle formation accompanied with increased carotenoid solubilization in intestinal fluids. Moreover, the boiling of tomatoes with emulsions increased carotenoid bioavailability compared to that observed when tomatoes were boiled alone and then added to emulsions [190].

Mehrad et al. [191] encapsulated β-Car into SLNPs containing palmitic acid and corn oil and stabilized by WPI, and the solid shell of the palmitic acid crystals formed on the surface of the oil droplets protected the encapsulated β-Car. Corn oil reduced the exclusion of β-Car from the solid lipid matrix to the surface of SLNPs, and WPI ameliorated β-Car oxidative stability. β-Car degradation increased with the increasing temperature and ionic strengths and decreasing pH. β-Car-loaded or β-Car and α-Toc co-loaded SLMPs prepared using palm stearin as the lipid phase and stabilized with a hydrolyzed soy protein isolate, which were incorporated in yogurt (5% of the total mass) did not affect the physicochemical or the rheological characteristics and the sensory quality of the product [192]. As in the previous section, of the mentioned nanoformulations, β-Car stabilization by WP [191] appears to be the most promising for strengthening of the immunity.

### 4.2. Coenzyme Q10

**Figure 4:**
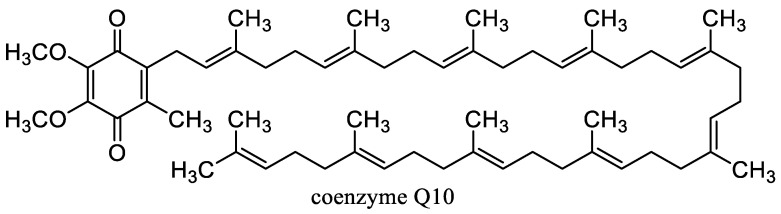
Structure of coenzyme Q10.

Coenzyme Q10 (ubiquinone; CoQ10, Figure 4) is benzoquinone, where Q is the quinone ring and the number 10 is the number of isoprenyl subunits (aliphatic chain) [193]. In 1978, Peter Mitchell was awarded the Nobel Prize in Research on CoQ10 [194]. CoQ10 is functionally similar to vitamins. It is found in most human cells except red blood cells and lenses. It contributes to the conversion of energy from food into chemical energy (ATP), so that CoQ10 can be found in the highest concentrations in the heart, lungs and liver. At the molecular level, it helps in the oxidoreduction of cytochrome bc1 in the electron transport chain on the mitochondrial membrane and also acts as an antioxidant in mitochondria and lipid membranes. CoQ10 is synthesized in the body but must also be ingested through food. The proportion of autosynthesis decreases with age, which may reduce the tissue concentration of CoQ10 [193,194]. CoQ10 prevents the aging process, has an antiatherogenic effect, and improves heart function. It has positive results against the manifestations of CNS dysfunction, increases the number and motility of sperm, and acts against wrinkles on the skin [194,195]. Administration of statins, antihyperlipidemics (inhibitors of 3-hydroxy-3-methyl- glutaryl-coenzyme A reductase, a key enzyme for the synthesis of endogenous cholesterol) also blocks endogenous synthesis of CoQ10 by the same enzyme [195].

The lipophilic nature and the large molecular weight of CoQ10 result in its low oral bioavailability. Current findings related to improvement of CoQ10 bioavailability in order to obtain its adequate intracellular and targeted mitochondrial delivery were presented by Zaki [196]. A review paper summarizing delivery systems for CoQ10 including liposomes, polymeric NPs, polymeric micelles, SLNPs, NLCs, self-emulsifying drug delivery systems, nanoemulsions, as well as solid and aqueous dispersions was presented by Kumar et al. [197].

By complexation of hydrophobic nutraceuticals such as CoQ10, CUR, and tocotrienol with γ-cyclodextrin (γ-CD), their bioavailability can be enhanced. Biologically active compounds, which dissociate from these insoluble complexes with γ-CD are captured by bile acid and form micelles without aggregation resulting in increased solubility and bioavailability [198].

RES and CoQ10 co-encapsulated in zein–propylene glycol alginate–rhamnolipid complex NPs showed better chemical stability under environmental stresses than the individual antioxidants entrapped in this carrier, and formulation exhibited also synergistical effect reflected in the improvement of the sustained release of RES and CoQ10 during in vitro digestion [199]. The co-encapsulation of CoQ10 and piperine into surface-modified core-shell NPs prepared by encapsulation of nutraceuticals in zein NPs followed by electrostatic deposition of κ-carrageenan on their surface and the subsequent inducing of κ-carrageenan layer into the hydrogel shell by using K^+^ as a crosslinking agent resulted in a 3.0- and 1.8-fold increase in photodegradation half-lives of CoQ10 and piperine compared to their free forms. Similarly, an increase in the retention rates of co-encapsulated nutraceuticals during thermal treatment (by 151% and 200%, respectively) or during four-week storage (by 111% and 131%, respectively) was observed. The release of nutraceuticals from the formulation in a simulated GI tract can be affected by the degree of interfacial cross-linking [200]. Triglyceride/phospholipid-based nanocarriers developed through high-pressure homogenization with the average particle size of 75 nm, showing the 95% EE of CoQ10, exhibited only a low leakage of CoQ10 in simulated GI fluids and the sustained release of the encapsulated nutraceutical [201].

Rhamnolipids were described as effective stimulators of immunity [202]; therefore, of the above described nanoformulations, it appears that RES and CoQ10 co-encapsulated in zein–propylene glycol alginate–rhamnolipid composite are the most advantageous for modulating the immunity [199].

### 4.3. Polyphenols

#### 4.3.1. Resveratrol

**Figure 5:**
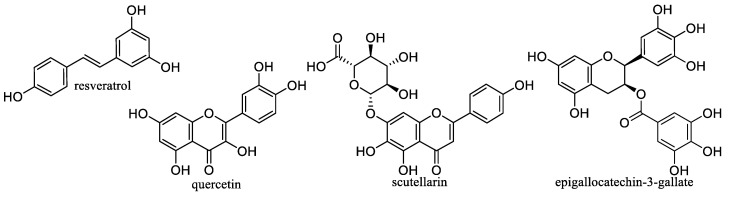
Structures of polyphenolic agents affecting immunity.

Resveratrol (RES, Figure 5) is a natural polyphenolic antioxidant derived from the structure of stilbene also exhibiting antibacterial effects and is therefore frequently used in food supplements. It occurs in a wide range of different vegetables and fruits [203,204]. It is believed that its ability to block androgen receptors has a positive effect on prostate cancer prevention [205]. Moreover, it has the potential to regulate blood pressure, modulate the immune system and energy metabolism, inhibit replication of human herpes viruses, influenza viruses, cytomegalovirus, and Epstein–Barr virus. On the other hand, it has a stimulating effect on other viruses, e.g., human hepatitis C virus [203,204].

The addition of Ca^2+^ at the dose of ≤4 mM enhanced the retention rate of encapsulated RES from ternary complex particles consisting of zein, propylene glycol alginate, and tea saponin and contributed to the improved sustained release of RES, the best results being observed with 2 mM Ca^+^. On the other hand, at the application of doses exceeding 6 mM, the aggregation of complex particles occurred [206]. RES-loaded zein–pectin core/shell NPs were stable to aggregation in a wide pH range (pH 2–7), showed good heat stability (80 °C for 1 h) as well as higher bioavailability, radical scavenging, and ferric ion reducing power than free RES, and powerful intracellular ROS scavenging was exhibited also by the GI fluids after digestion of the encapsulated RES [207]. Spherical RES-loaded PPI NPs prepared using Ca^2+^ as a cross-linking agent, showing the EE of 74.08% and physical stability, ensured the superb protection of RES from degradation and markedly improved its antioxidant ability [208].

Spherical α-lactalbumin-RES NPs showed 32-fold higher water solubility and in vitro antioxidant activity than free RES and improved the chemical stability of RES under storage, especially at pH 8.0 and high temperature [209]. RES-loaded ovalbumin–carboxymethylcellulose (OVA–CMC) NPs fabricated by heating OVA–CMC nanocomplexes at 90 °C for 30 min showed the EE of ca. 70% and 35 g/mg loading capacity, improved the photostability of *trans*-RES under exposure to UV light, and ensured 80% bioavailability of RES, suggesting that such NPs can be used for effective oral delivery of RES [210].

Freeze-dried RES-loaded gliadin NPs stabilized by GA and CS HCl fabricated using a gliadin/GA/CS HCl ratio of 1:2:1 and gliadin/RES ratio of 8:1, with a mean particle size of ca. 300 nm and EE 68.2%, exhibited markedly improved RES release in simulated GIT conditions and higher Fe^3+^ reducing antioxidant power than RES-loaded gliadin particles, while RES-loaded gliadin-GA NPs exhibited stronger DPPH radical scavenging activity [211]. Nanocapsules fabricated by the electrostatic deposition of CS onto the negatively charged core consisting of a cyclodextrin metal–organic framework showed higher EE for RES than nanocapsules without CS coating (90.3% vs. 66.5%) and enhanced the antioxidant activity and the photostability of the encapsulated RES [212].

RES encapsulation in NEs prepared using an orange oil-to-grape seed oil ratio of 1:1 (*w*/*w*) and Tween 80 surfactant by spontaneous emulsification with the mean droplet size of ca. 100 nm showed improved chemical stability of RES after exposure to UV-light, achieving 88% retention compared to 50% observed in dimethylsulphoxide [213]. RES-loaded SLNPs and NLCs with particle sizes of 150–250 nm, zeta potential ca. −30 mV, and EE 70% showed good stability over 2 months. The investigation of the in vitro simulation of GI transit showed that the RES remained mostly associated with the lipid NPs after their incubation in digestive fluids [214]. The addition of niosomes incorporating RES, which were prepared using Span^®^ 60 or Maisine^®^ 35-1 as surfactants, and dodecanol as stabilizer, to yoghurt did not affect its textural properties suggesting that they can be used as additives in this dairy product [215].

RES-loaded nanofibers with zeta potential ranging from −20.5 to −32.2 mV, average fiber diameters 63–208 nm, and EE 74 and 96.70% prepared by electrospinning at applied voltages of 18 and 23 kV were characterized with high stability and maintained RES antioxidant activity, and their use for milk fortification did not result in considerable physiochemical and sensorial changes [216].

As it was found that α-lactalbumin has certain benefits for immunity [217], the α-lactalbumin–RES nanoformulation [209] can be considered as particularly advantageous of the mentioned nanosystems.

#### 4.3.2. Catechins

Quercetin (QR, Figure 5) is a polyphenolic flavonoid, which is found in many fruits, vegetables, leaves, seeds and grains. It has a bitter taste and is used as an ingredient in food supplements, beverages, and food. QR is one of the most abundant dietary flavonoids with an average daily intake of 25–50 mg. QR is in fact an aglycone of many flavonoid glycosides, such as rutin or quercitrin. The bioavailability of QR is low and highly variable (0–50%). It is rapidly eliminated (half-life is about 1–2 h) and is metabolized very rapidly after ingestion, making it unlikely that the biological effects expected from in vitro studies would be administered in vivo. QR has been reported to inhibit the oxidation of other molecules, thus acting primarily as an antioxidant, a free radical scavenger. QR also activates or inhibits a number of enzymes. QR has been studied as an adjunct in the treatment of cancer and various other diseases [218,219,220].

As convenient lipid-based delivery systems for QR, which improve its stability and bioavailability, NEs fabricated using high pressure homogenization were also reported [221]. Comparison of SLNPs, NLCs, and lipid NEs from the aspect of QR delivery showed that the particle size of nanocarriers increased in the following order: NLCs (≈34–47 nm) < lipid NE (≈82–83) < SLNPs (≈103–127 nm). All tested nanocarriers showed >90% EE for QR and the bioavailability at incubation in simulated GI conditions decreased as follows: NLCs and NEs (≈60%) > SLNPs (≈35%) > free QR solution (≈7%). Controlled release in enzyme free simulated intestinal fluid with maximum release was observed with NEs [222]. Liposomes prepared with unsaturated egg Lipoid^®^ E80 used as phospholipid showed higher QR EE and ensured better protection of QR against UV irradiation than those fabricated using unsaturated soybean Lipoid^®^ S100 and saturated soybean Phospholipon^®^ 90H, likely due to their different membrane rigidity. The liposome formulations fabricated with all three of the above mentioned phospolipides were physically stable after 1 year of storage at 4 °C, and by the encapsulation of a sulfobutylether-β-cyclodextrin/QR inclusion complex in Lipoid^®^ E80 liposomes, an additional improvement of QR photostability could be achieved [223].

The polymeric niosomes composed of hyaluronic acid and QR had the size of 231.07 ± 8.39 nm and the zeta potential of −34.00 ± 0.95 mV. The hyaluronic acid scavenging efficiency was 94.67 ± 1.62%. The DPPH experiment showed that 80 µL of niosomes (7.46 × 10^−8^ mol of quercetin) had a significant antioxidant effectiveness. The efficacy of the nanoformulation in suppressing inflammation (edema formation) in rats after oral administration was superior to that of the bulk QR suspension [224]. LbL composite NPs prepared from zein and hyaluronic acid (HA) encapsulating CUR and quercetagetin (mean particle diameter: 231.2 nm, zeta potential: −30 mV) with EE 69.8 and 90.3%, respectively, were found to be stable over 6 months of storage ensuring the superb protection of both nutraceuticals against photodegradation and thermal degradation. With increasing HA concentration in the composite NPs, the morphology of NPs was changed from coated NPs nanoparticles to NPs-filled microgels. Under simulated GI conditions, this nanoformulation released CUR and quercetagetin slowly, suggesting the improved oral bioavailability of nutrients entrapped in above mentioned LbL composite NPs [225]. Casein NPs and re-assembled casein micelles (r-CMs) markedly improved the chemical stability of encapsulated QR and increased its aqueous solubility compared to free QR, and the viability of MCF-7 human breast cancer cells in the presence of digested QR-loaded r-CMs was lower than that of non-digested QR-loaded carriers and free QR [226].

Epigallocatechin-3-gallate (EGCG, Figure 5) catechin polyphenol found in green tea leaves, is an ester of epigallocatechin and gallic acid. Orally administered EGCG has poor absorption. EGCG has shown various biological effects, e.g., it is a modulator of the GABA_A_ receptor and can lower LDL cholesterol [227,228,229]. In their review paper, Granja et al. [230] focused on the therapeutic potential of EGCG nanodelivery systems. The addition of EGCG to β-glucan stabilized virgin coconut oil NEs contained in surimi gels markedly improved the antioxidant activities and the antidiabetic activity of the surimi digest, while reducing its angiotensin-converting-enzyme inhibitory activity [231]. By adding EGCG to pre-heated (75–85 °C, 20 min) β-lactoglobulin solution during cooling and vortexing, thermally-induced protein–EGCG co-assemblies with sizes <50 nm effectively protecting EGCG against oxidative degradation were obtained [232]. Glycated WPI–EGCG nanocomplex-stabilized emulsion encapsulating β-Car was characterized with improved storage, salt ion, and thermal stability compared to the non-emulsified nanocomplex and was able to markedly inhibit the degradation of β-Car. On the other hand, due to the powerful hydrolysis of WPI by pancreatin, the emulsion cannot survive in the intestinal fluid resulting in faster and more intense lipolysis compared to the naked MCT [233].

Scutellarin (SCL) is a flavone that can be found, for example, in *Scutellaria lateriflora* and *S. barbata*. SCL showed anti-HIV and anticancer effects. However, its most important indication is the treatment of diabetic retinopathy [234,235,236]. The limitation is its low bioavailability. Therefore, Wang et al. prepared a nanoformulation of SCL encapsulated in CS modified by deoxycholic acid and vitamin B_12_ (CS-DS-VB_12_). CS-DC-VB_12_-SCL NPs were spherical with a particle size ranging from 150 to 250 nm and had high permeation through Caco-2 and low toxicity against Caco-2 cells at the concentration 250 μg/mL. Subsequent in vivo bioavailability tests performed on Sprague–Dawley rats showed an area under the SCL curve of CS-DC-VB_12_-SCL NPs two to three-fold larger than that of bulk SCL. Furthermore, retinal neovascularization via the down-regulated expression of angiogenesis proteins (VEGF, VEGFR2 and vWF) in type II diabetic rats was also inhibited [237].

From the above-mentioned nanoformulations of catachins, the combination with β-glucan [80,231] appears to be the most beneficial for enhancing human immunity.

### 4.4. Curcumin

**Figure 6:**
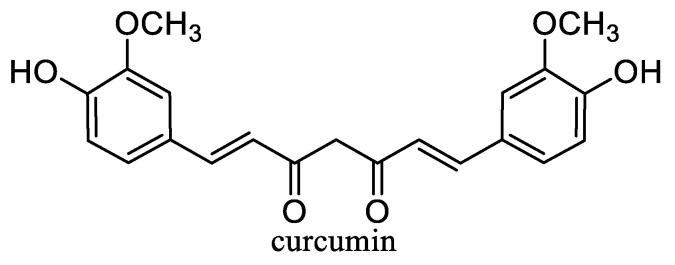
Structure of curcumin.

Curcumin (CUR, Figure 6) is a yellow to orange natural dye extracted from the rhizomes of plant species of the genus Turmeric, especially *Curcuma longa* or *Curcuma zedoaria* (*Gingiberaceae*). CUR is a derivative of the basic molecule 1,7-bis(4-hydroxy-3-methoxyphenyl)hepta-1,6-diene-3,5-dione. In the food industry, it is marked with the code E 100. CUR is mostly used as a colorant and antioxidant in food products. In folk medicine, a decoction of ground drugs is used for postpartum abdominal pain and menstrual arrest, GI disorders (bloating, colic), kidneys, liver, and gallbladder. It is applied externally as a disinfectant solution or as a dusting for non-healing ulcers, lichens and various skin injuries. Beside strong antioxidant effects, CUR has also anti-tumor and anti-inflammatory effects in vivo, lowers cholesterol levels, and nanosized CUR can have a prophylactic effect on Alzheimer’s disease [238,239,240].

Kotha and Luthria [241] summarized recent research on biological, pharmaceutical, and analytical aspects of the CUR. Anticancer, antioxidant and antiangiogenic activities of NPs fabricated of bioactive dietary nutraceuticals with beneficial impact on the immunity including CUR, QR, and flavone was discussed by Bansode et al. [242]. Nanoformulations capable to enhance CUR oral bioavailability through nanoformulations were comprehensively overviewed by Ipar et al. [243], and findings related to CUR delivery mediated by bio-based NPs were summarized by Nasery et al. [244]. Using CUR as a model nutraceutical, Kharat and McClements [245] analyzed the potential of the delivery by the design approach for identifying and selecting colloidal delivery systems that are most favorable for a particular food application.

Zheng et al. [246] used the pH-shift method for enrichment of bovine milk with CUR without adversely affecting milk fat globule stability. CUR-enriched milk showed pH stability at pH 6.5, 7.0, and/or 8.0 during storage at 4 °C lasting 60 days, whereby CUR breakdown decreased with decreasing storage temperature from 43% at 55 °C to 5% at 4 °C. Concerning the incubation of the milk up to 37 °C, its color practically did not change. Under in vitro GIT conditions, the CUR bioavailability was approx. 40%.

In an experiment, in which powdered CUR was dispersed into the O/W NEs using the conventional oil-loading method (60 °C, 2 h), the heat-driven method (CUR added to NE and then incubated at 100 °C for 15 min), and the pH-driven method (CUR dissolved in a solution of pH 12.5 added to an acidified NE of pH 6.0), the highest EE of CUR (93%) was observed using the pH-driven method. Although the CUR bioavailability from the above three formulations was similar (74–79%), the highest amount of CUR in the mixed micelle phase was observed also with the NE fabricated by the pH-driven method [247]. O/W NEs encapsulating CUR prepared using sodium caseinate or sodium caseinate and pea protein isolate mixture (1:1) released ca. 50% CUR in the simulated intestine phase, although the lipid digestibility of the NE fabricated using the mixture of proteins was markedly lower compared to that prepared exclusively with sodium caseinate. The presence of digestive enzymes and rapid changes in the ionic strength and pH during in vitro digestion resulted in an increase in droplet sizes and changes in their distribution [248]. The EE of CUR and QR by casein NPs as well as re-assembled casein micelles exceeded 90%, and the chemical stability of phenolic compounds was markedly enhanced compared to the free compounds; loading of both tested antioxidants into re-assembled casein micelles increased their solubility [226]. By in situ cross-linking of CUR with collagen, aerogels were developed showing strong proteolytic and anti-microbial activity, whereby their 3D microstructure enabled higher adhesion and proliferation of cells and increased the permeability and water-retaining ability inevitable for the diffusion of the compound supporting cellular growth. Due to superb pro-angiogenic efficacy of these collage aerogels, in which CUR was used as a cross-linker, they can be also used for biomedical applications [249].

CUR encapsulated in sunflower seed protein isolate NPs showed increased solubility and strong antioxidant activity, and its anti-inflammatory activity estimated based on lipoxygenase inhibition achieved the IC_50_ of 45.3 µM. The nanoformulation was stable in water and in GI conditions [250]. CUR encapsulated in lactoferrin nanohydrogel was bound to lactoferrin via hydrophobic interactions, and the formulation was stable up to 35 and 14 days of storage at 4 °C and 25 °C, respectively. The release rates of CUR from lactoferrin hydrogel were 1.75-fold higher in a food simulant of lipophilic nature (50% ethanol) than in that of hydrophilic nature (10% ethanol). The incorporation of lactoferrin nanohydrogels into a gelatine matrix was not accompanied with their degradation [251].

Anionic spherical core-shell NPs (<200 nm) with a core consisting of zein–epigallocatechin gallate conjugates and a rhamnolipid shell encapsulating CUR and RES protected them from degradation, whereby their antioxidant activity was preserved. At co-encapsulation of both antioxidants their loading efficiencies were enhanced compared to that of individual compounds. At the application of a mixture of core-shell NPs with lipid droplets, the bioavailability of both CUR and RES was markedly ameliorated [252]. Ternary complexes consisting of protein (zein), polysaccharide (propylene glycol alginate) and surfactant (either rhamnolipid or lecithin) used as a delivery system for CUR showed >90% EE of CUR and considerably increased its photostability and bioavailability [253]. Chen et al. [254] fabricated core-shell NPs using zein as the core and a ι-carrageenan as the shell for the co-encapsulation of CUR and piperine protecting both nutraceuticals from the photodegradation and thermal degradation, in which the outer layer was cross-linked using Ca^2+^ ions. The core-shell NPs delayed the release of encapsulated compounds under in vitro GI conditions, resulting in their ameliorated oral bioavailability. Huang et al. [255] produced a shell around zein NPs using a combination of ALG showing high charge density and pectin showing low charge density, in which a 30% ALG content markedly ameliorated aggregation stability in the pH range 5–7 and at high ionic strength (2000 mM NaCl). CUR encapsulated in these NPs showed higher antioxidant and radical scavenging activities than free CUR. CS and ALG layers’ deposition on CUR NEs ensured the improved control of the rate and the extent of lipid digestibility due to reduced FFAs release compared to uncoated NEs and ameliorated CUR antioxidant capacity during in vitro digestion, although CUR bioavailability was found to be lowered [256]. An oligo-hyalurosome nanodelivery system fabricated by loading CUR and RES into an oligo-hyaluronic acid–CUR polymer with mean particle size 134.5 ± 5.1 nm and zeta potential −29.4 ± 1.2 mV under pH 7.4 phosphate-buffered saline conditions exhibited superb stability, a dose-dependent GI release of antioxidants in vitro, and higher radical scavenging activity compared to the single formulations and liposomes, suggesting that this nanodelivery system could be successfully used in juice, yoghourt, and nutritional supplements [257].

A CUR–β-cyclodextrin inclusion complex and iron oxide NPs co-encapsulated within liposomes showing sizes of 67 nm and 71% EE of CUR synergistically enhanced radical scavenging properties compared to that of conventional curcumin liposome and iron oxide NPs [258].

Sophorolipid-coated CUR NPs (61 nm; zeta potential: −41 mV; EE: 82%) showed 2.7–3.6-fold higher bioavailability both in vitro and in vivo compared to free CUR, which was connected with their higher bioavailability [259]. Saponin-coated CUR NPs prepared by a pH driven loading method were found to be aggregated at low pH values (<3) and high NaCl concentrations (>200 mM), while they were stable in a wide pH range (pH 3–8) and lower salinity (<200 mM NaCl) as well as during refrigerated storage at 4 °C or after conversion into a powdered form (lyophilized). These NPs showed approx. 3.3-fold higher in vitro bioavailability and 8.9-fold higher in vivo bioavailability after oral administration to Sprague–Dawley rats than free CUR [260]. The modification of CUR liposomes with Pluronic^®^ F127, F87, and P85 enhanced their pH stability and thermal stability and resulted in a slower release rate, a lower cumulative release percentage for CUR. Moreover, in vitro simulated GIT studies showed that such modification considerably improves the absorption of CUR liposomes, the best CUR bioavailability being observed with Pluronic^®^ F127 [261].

Based on the above listed nanoformulations, it can be stated that the combination of curcumin and sodium caseinate [226] appears to be the most effective for strengthening immunity.

## 5. Fatty Acids

**Figure 7:**
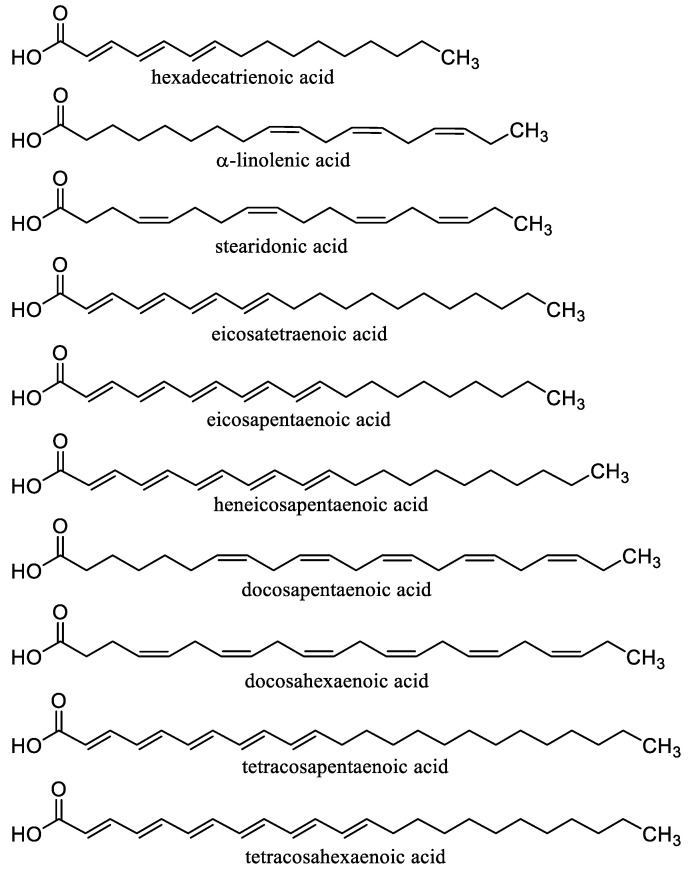
Structures of polyunsaturated omega-3 fatty acids.

Omega-3 unsaturated fatty acids, such as α-linolenic acid or docosahexaenoic acid belonging to polyunsaturated fatty acids (PUFAs, Figure 7), is a group of unsaturated fatty acids, the common feature of which is the double bond between the carbons in the third and fourth positions. PUFAs cannot be synthesized in the mammalian body, but are important for human metabolism [262]. Their effect on the cardiovascular system is complex; PUFAs regulate the rheological properties of the blood, regenerate the vascular endothelium, stabilize the myocardium, reduce the extent of ischemic damage, and positively affect the metabolic syndrome (hypolipidemic effect, glycemia regulation) [263,264]. Omega-3 PUFAs have an anti-inflammatory effect, improve the immune system, have a positive effect on the development of the CNS and memory, and reduce the risk of depression [262,263,264].

For administration of omega-3 PUFAs in combination with other nutraceuticals or with conventional/innovative drugs, nanoformulations could be used ensuring protection of PUFAs from degradation, improving their bioavailability, enabling their delivery to target tissues resulting in enhanced bioactivity, and such formulations are particularly prospective for application in cardiovascular and neoplastic diseases [265]. Valenzuela et al. [266] discussed the advantages of marine phospholipids compared to triglycerides as a source of omega-3 PUFAs considering that these phospholipids from marine origin contain high concentrations of docosahexaenoic acid (DHA) and show higher bioavailability and resistance to oxidation than triglycerides.

The fabrication of NE-based delivery systems for oils containing omega-3 PUFAs using a spontaneous emulsification method, which could be applied in clear foods, was described by Gulotta et al. [267]. Quillaja saponin, a biosurfactant of natural origin characterized with high free radical scavenging capacity, applied to prepare an NE encapsulating omega-3 oils using high pressure homogenization (microfluidization) was found to protect the encapsulated compounds against oxidation more efficiently than other surfactants such as lecithin, Tween 80, and sodium dodecyl sulfate [268]. NEs fabricated using lemon oil and thyme oil containing high levels of phenolics showing antioxidant activity used for the encapsulation of fish oil protected it from oxidation much more effectively than NEs, in which MCT were used as a carrier oil. The best protection against oxidation was provided by NEs with thyme oil. All prepared NEs consisting of 75% fish oil and 25% carrier oil were found to be physically stable during storage at 20 °C for 42 days [269].

Food-grade NEs (mean particle size of 52.3 nm) prepared using edible biopolymers CS and gelatin at a ratio 90:10 as wall materials with encapsulated unsaturated fatty acids concentrates from carp oil showed high stability after 7 days of storage (peroxide value of 4.8 meq/kg, *para*-anisidine value of 9.8/meq kg, and Tox value of 19.4 meq/kg) [270]. The fish oil formulated in NEs exhibited a markedly higher rate of uptake of PUFA in three segments of small intestine, and its bioavailability was positively correlated with its inhibitory response against lipopolysaccharide-induced NO production in rat peripheral blood mononuclear cells (PBMCs) [271]. Fish oil NEs with soybean protein isolate-phosphatidylcholine (SPI-PC) showed better storage and oxidative stability and had better resistance to salinity at 0.1–0.5 M of Na^+^ but lower resistance to acidic conditions than the NE prepared with Tween 20, and NE droplets aggregated during in vitro gastric digestion in contrast to the NE fabricated with Tween 20. On the other hand, the use of SPI-PC NEs markedly improved the digestibility of fish oil, whereby even 86.8% release of FFAs was observed in the 2-h in vitro intestine digestion [272].

A WPC multiple NE with sizes of 190–210 nm encapsulating fish oil ensured the oxidative stability of incorporated compounds, which increased with increasing concentrations of WPC, and this effect could be intensified by co-encapsulation of vitamin C [273]. WPC was used as a matrix for encapsulation of nanodroplets of DHA-enriched algae oil using emulsion electrospraying assisted by pressurized gas, whereby the oil was situated in nanometric cavities (<300 nm) of fabricated particles (ca. 5 μm). The encapsulated oil was protected against oxidation, and the oil encapsulation practically did not affect its organoleptic properties in milk powder. On the other hand, when maltodextrin was used as a matrix instead of WPC, the secondary oxidation could not be excluded [274]. Electrosprayed ultrathin capsules of zein encapsulating DHA reduced the DHA degradation rate; they were relatively stable at changing relative humidity and temperature, and encapsulated DHA released considerably lower amounts of three main flavor-influencing aldehydes [275].

The NE formulation fabricated using 0.5% (*w*/*v*) of sesame protein isolate as a natural surfactant in combination with Tween 20 and Span 80 (1:1) showing droplet sizes up to 89.68 nm and shelf-life stability up to 8 weeks with encapsulated omega-3 PUFA rich fish oil released ≥90% of fatty acids during 120 min of simulated two-step in vitro digestion and did not show toxicity to rat PBMCs [276].

Casein showing significant affinity to DHA (K_b_ = 8.38 ± 3.12 × 10^6^ M^−1^) was reported to bind ca. 3–4 DHA molecules per protein molecule. DHA-loaded casein NPs with a diameter of 288.9 ± 9.6 nm as well as DHA-loaded re-assembled casein micelles prepared by the addition of Ca and PO_4_^3−^ with sizes of 50–60 nm excellently protected DHA against oxidation, were characterized with satisfactory stability, and maintained biological activity at 4 °C, indicating their suitability for the enrichment of foods and beverages [277]. Nanosized complexes formed between a covalent conjugate consisting of sodium caseinate and maltodextrin and combinations of polyunsaturated lipids (linoleic acid + α-linolenic acid (α-LA), liposomes of soy phosphatidylcholine + α-LA, and micelles of soy lysophosphatidylcholine + α-LA) were characterized with high EE (>95%) and provided not only superb protection of the lipids but also good solubility in an aqueous medium [278].

The high EE of DHA in nanocomplexes of β-lactoglobulin with pectin was reported, whereby DHA was found to be effectively protected against degradation, and over 100 h at 40 °C only 5–10% DHA were lost from the formulation compared to 80% observed with free DHA, suggesting that the nanocomplexes of β-lactoglobulin with pectin could be utilized for enrichment of clear acid drinks with DHA [279]. Direct spray drying applied for the microencapsulation of DHA in hydroxypropyl methylcellulose acetate succinate was found to be favorable for achieving high EE and DHA stability in microcapsules, and rapid release in phosphate buffer (pH 6.8) was observed after the lag time of 2 h in acidic media [280].

In NLCs consisting of Poloxamer 407 and cocoa butter (solid lipid) and conjugated linoleic acid (liquid oil) in a ratio of 10:1, the omega-3 PUFA was protected against oxidation at the level of 3.9% of milk fatty acids for 40 days when formulation was kept at 4 and 22 °C [281]. A hydrodynamic flow focusing polyimide microfluidic device used to fabricate hexosomes (108–138 nm) based on DHA monoglyceride in the presence of the stabilizer Pluronic^®^ F127 was described by Yaghmur et al. [282]. Such hexosomes may be utilized for the delivery of omega-3 PUFAs, drugs, nutraceuticals, and their combinations.

Shao et al. [283] investigated structural characteristics of self-assemblies based on monoglycerides of DHA and docosapentaenoic acid at exposure to excess water and found that both lipids tend to form a dominant inverse hexagonal (H_2_) phase at 25 °C and a temperature-triggered structural transition to an inverse micellar solution (L_2_ phase). Such NPs can utilize the health promoting impact of omega-3 PUFA monoglycerides and combine it with that of loaded therapeutic agents or nutraceuticals. Furthermore, the application of nanoliposomes and tocosomes (a bioactive carrier made mainly from tocopheryl phosphates), which can carry separately or simultaneously hydrophilic and hydrophobic cargo and provide protection and release of encapsulated nutraceuticals and dietary molecules in health promoting dietary supplements and functional foods, was reviewed by Zarrabi et al. [284].

As mentioned above [123], the combination of casein and PUFAs appears to be the most promising to improve the immunity [277].

## 6. Probiotics

Probiotic products contain living microorganisms that have beneficial effects on the health of the consumer by improving the balance of his intestinal microflora. Classic examples of foods containing probiotics are yogurt, acidophilic milk, and kefir. The most common probiotics are lactic bacteria of the genera *Lactobacillus*, *Bifidobacterium* and several other bacteria and yeasts, such as *Enterococcus faecium*, *Streptococcus thermophilus*, and *Saccharomyses cerevisiae*. Probiotics are thought to support the natural metabolism and function of the GIT, prevent the formation of toxins, promote immunity, healing, and alleviate the side effects of certain drugs [285,286,287,288].

Microencapsulation is the most widely used technological modification of lactic acid bacteria in the food industry to improve their viability and survival in the GI milieu of hosts [289]. The potential use of lactic acid bacteria as starter culture in fermented foods with comprehensive overview of respective coating materials and factors affecting encapsulation was discussed by Kavitake et al. [290]. Advantages of microencapsulation of probiotic bacteria related to enhancing the viability during processing and targeted delivery in GIT were already summarized by Anal and Singh [291]. A review paper of Kwiecien and Kwiecien [292] focused on polysaccharide (CS, ALG, κ-carrageenan, xanthan, and pectin) hydrogels consisting of one or more polysaccharides used as probiotic delivery systems improving the viability of probiotics in GIT and during storage and thermal processing. In addition, by nanoencapsulation of probiotics, their bioavailability, stability, and viability can be improved [293]. Durazzo et al. [294] in their review paper comprehensively summarized the findings related to the production and application of nanonutraceuticals with emphasis on nanoprebiotics and nanoprobiotics. Nanoformulations suitable for the encapsulation of probiotic bacteria include NPs, nanofibres, nanobeads, nanolayers fabricated by the LbL technique, as well as NEs. The LbL encapsulation of probiotics for delivery to the microbiome with emphasis on the protection of probiotics at acidic gastric conditions and improvement of their adhesion and growth in the intestines was discussed by Anselmo et al. [295].

Advantages of individual microencapsulation techniques in ensuring the survival of encapsulated probiotic bacteria during processing, storage, and GI digestion were summarized by Liu et al. [296]. The researchers discussed currently applied high temperature drying technologies such as spray drying and fluid bed drying as well as those of low temperature drying such as ultrasonic vacuum spray drying, spray chilling, electrospinning, supercritical technique, freeze drying, extrusion, emulsion, enzyme gelation, and the impinging aerosol technique. For example, *Saccharomyces boulardii* and *Enterococcus faecium* microencapsulated using emulsion and internal gelation in beads with sizes of 300–500 μm showed survival rate increases by 25% and 40%, respectively, compared to free cells under high temperature and high humidity. Encapsulation also increased the survival rate in SGF and SIF by 60% and 15%, respectively, for *S. boulardii* and by 25% and 20%, respectively, for *E. faecium* [297].

The survival of *Bifidobacteria longum* Bb-46 microencapsulated in alginate microspheres (20–70 μm) was improved over that of free cells during refrigerated storage in milk with 2% fat, although the presence of the encapsulated formulation in milk resulted in off-flavors not found in samples with free cells suggesting altered metabolism of encapsulated cells. The highest resistance to low pH and bile from nine tested bifidobacteria was observed with encapsulated formulation of *Bifidobacteria lactis* Bb-12 [298]. *Bifidobacterium* BB-1 encapsulated in MPs fabricated by emulsification using Na-ALG as wall material showing a slow release of Ca^2+^ ions was found to be resistant to the simulated GI conditions; the MPs were stable in buffer with pH 4.5, and total release of probiotics was observed at pH 7.5. After 120 days of storage, best viability was obtained at frozen storage (−18 °C), while storage at higher temperatures (7 °C and 25 °C) was accompanied with loss of the viability of encapsulated bacteria [299]. Highly acid-sensitive *Bifidobacterium adolescentis* (ATCC 15703) incorporated in legume protein–ALG capsules released almost all cells in SIF, regardless of the wall material, after the first 10 min. Encapsulated *B. adolescentis* cells were able to survive in pineapple and white grape juice, but not in orange juice [300].

For the microencapsulation of *Lactobacillus* *paracasei* A13 and *Lactobacillus salivarius* subsp. *salivarius* CET 4063, Patrignani et al. [301] applied Na-ALG, vegetable oil, and high pressure homogenization (50 MPa). The prepared microcapsules with incorporated microorganisms (<100 μM) were used to produce functional fermented milks. The supplementation of such microcapsules decreased the hyperacidity phenomena caused by the addition of free probiotic microorganisms in fermented milks, which resulted in ameliorated viability of microorganisms and improved sensory characteristics of the products. Moreover, encapsulated *Lactobacillus* strains showed improved resistance to the simulated digestion processes and higher resistance to the gastric barrier, observed with the *L. paracasei* A13 strain. On the other hand, microencapsulation resulted in reduced production of exopolysaccharides, and the modification of the volatile profiles compared to free microorganisms was observed. The protection of probiotic strains *Pediocucus acidilactici*, *Lactobacillus reuteri*, and *Lactobacillus salivarius* incorporated in ALG beads was improved by adding inulin, and the application of 5% *w*/*v* inulin ensured the highest bacterial protection against bile-salts [302]. *Lactobacillus plantarum* microencapsulated in Na-ALG or in Na-ALG-citric pectin matrices showed increased cell viability under refrigeration storage for 21 days as well as survival in the digestive system of hosts [303]. *Lactobacillus acidophilus* microencapsulation in ALG-gelatin as well as ALG-gelatin-fructooligosaccharides showed positive effect on the probiotic survival in yogurt during the storage compared to free *L. acidophilus*; the encapsulated *L. acidophilus* was found to be protected during storage and in simulated GI conditions, whereby the application of fructooligosaccharides contributed to gradual and controlled delivery along the GIT [304]. *Pediococcus pentosaceus* Li05 probiotic encapsulated in ALG-gelatin microgels doped with MnO NPs was found to be more stable than free bacterial cells or those encapsulated in microgels alone, whereby MnO NPs also increased the viability of *P. pentosaceus* Li05 by filling pores inside the microgels and in a such way hindered the access of O_2_ and H^+^ ions to the probiotic. Moreover, the neutralization of H^+^ ions in the gastric fluids by MgO NPs contributed to the reduced acid-induced degradation of the probiotics [305]. The viability and storage stability of *L. acidophillus* microencapsulated in ALG together with the prebiotics rice bran, inulin, and hi-maize were better than those of free cells, and these formulations also provided better protection for *L. acidophillus* under simulated GI conditions [306]. The addition of cellulose nanocrystal and lecithin in ALG microbeads encapsulating *Lactobacillus rhamnosus* ATCC 9595 improved its viability during gastric passage by 37% as well as during storage at 25 and 4 °C compared to neat alginate microbeads [307].

Multilayered microcapsules consisting of *L. acidophilus* encapsulating ALG microcapsules (EE >90%) coated with a composite of egg albumin and stearic acid (first layer) and subsequently coated with cassava starch were designed by Pitigraisorn et al. [308]. They were able to provide very high protection of the encapsulated microbial cells exposed to moisture and heat, when loss of vitality achieved only 0.6 log_10_(CFU/g), suggesting that they are suitable to be used for the protection of thermosensitive microorganisms and compounds applied for the fortification of foods and feeds. It could be noted that the survival rate of probiotics entrapped in microcapsules exposed to the moist-heat processing can be markedly ameliorated by increasing the relative proportion of stearic acid.

CS-coated ALG microcapsules encapsulating *Bifidobacterium longum* protected *B. longum* from GI fluid and heat stress and showed improved heat, acid, and bile salt tolerance and stability as compared to the uncoated microcapsules, which was reflected in the lower reduction of viable bacterial cells [309]. Improved survival during gastric transit and storage was shown also by *Bifidobacterium bifidum* encapsulated in zein-coated Na-ALG hydrogels [310]. *Lactococcus lactis* ssp. *cremoris*, which is able to produce 952 ± 26 μg/L of folate during 10 h after its encapsulation into ALG/ε-poly-l-lysine/ALG/CS microcapsules, maintained the viable concentration of 6 log_10_(CFU/mL) under simulated digestion conditions. Coated microcapsules were found to adhere to epithelial cells and were able to exchange nutrients, allowing probiotic activation [311]. The physiological protection of probiotic microcapsules by coatings was discussed by Ramos et al. [312], who highlighted that CS, ALG poly-l-lysine, and WP could provide excellent protection against the harsh conditions of digestion and emphasized that the use of several coatings may not result in better protection than that observed with monocoated microcapsules.

The beneficial impact of CS-coated microcapsules on the survival rates of different probiotic bacteria under in vitro GI conditions and the storage stability of different types of food products was summarized by Calinoiu et al. [313]. The encapsulation of *L. acidophilus* by CS decreased the leakage of probiotic bacteria when compared with free bacteria; the reduction of bacterial count in gastric acid was relatively low, and the formulation provided desirable probiotic viability and stability in intestinal juice [314]. The presence of CaCO_3_ in capsules fabricated by ionic gelation between phytic acid and CS, using the electrostatic extrusion method and CaCO_3_ and starch co-encapsulants, which incorporated *L. acidophilus*, protected the encapsulated bacteria against acid injury via antacid and buffering effects [315]. *L. acidophilus* encapsulated in xanthan–CS and xanthan–CS–xanthan polyelectrolyte complex gels by the extrusion method showed markedly higher resistance to both SGF and SIF compared to free bacteria. Encapsulation also considerably improved the survival of bacterial cells in yogurt during 21 days of storage at 4 and 25 °C compared to free cells [316]. *L. acidophilus* cells incorporated into multilayer capsules fabricated via the LbL self-assembly of CS/CMC showed survival corresponding to 33 log% of the cells when exposed to SGF and subsequently to SIF for 120 min, while approx. complete death of free cells was observed at these conditions, which was connected with the impermeability of applied polyelectrolyte nanolayers to pepsin and pancreatin causing proteolysis, which ameliorated the stability of coatings at gastric and intestinal pH; the formulation showed increased stability also during freezing and freeze-drying compared to free cells [317].

*Lactobacillus casei*, *Lactobacillus brevis*, and *L. plantarum* encapsulated in β-glucan matrix using emulsion technique showed markedly improved tolerance against abiotic stresses (low pH, heat processing), simulated GI conditions, and storage [318]. Phase-separated gelatin–maltodextrin microspheres with the maltodextrin core and an outer gelatin layer cross-linked with transglutaminase able to withstand pepsin-induced degradation in SGF (pH 2.0, 2 h, 37 °C) with encapsulated *Lactobacillus* sp. situated predominantly in the gelatin–maltodextrin interphase improved the survival of microorganisms in SGF [319].

The comparison of microcapsules of inulin, hi-maize, and trehalose encapsulating *L. acidophilus* La-5 fabricated by spray drying showed that the best EE was achieved using inulin and hi-maize as encapsulated matrices, while the greatest thermal resistance and storage stability of 120 days were shown by the trehalose matrix. Although the greatest viability of *L. acidophilus* La-5 in simulated GI conditions was observed with hi-maize encapsulating matrix, all three tested matrices were able to keep the counts of microorganism above the recommended level, suggesting superb protection of *L. acidophilus* La-5. This was supported by the fact that no ruptures or cracks on the surfaces of the MPs were estimated [320].

An O/W NE consisting of coconut oil and WPC, inulin, and GA in the aqueous phase was fabricated by Krithika and Preetha [321]. After adding the *Enterococcus faceium* probiotic organism into the aqueous phase, the NE showed an increase in particle size from <150 nm up to 500 nm after 60 days of storage but maintained the viability of *E. faceium*, suggesting the suitability of the WPC/inulin nanocomplex to be used as a delivery system for probiotics in food products. Probiotic bacteria *L. acidophilus* Ki, *L. paracasei* L26, and *Bifidobacterium animalis* BB-12 microencapsulated by WP containing l-cysteine-HCl protected probiotic cultures from simulated GI conditions. In contrast to *B. animalis* BB-12 and *L. acidophilus* Ki, which were found to be susceptible to abiotic stresses, *L. paracasei* L26 protected in WP microcapsules stored at 22 °C for 180 days showed >10^6^ CFU/g, irrespective of relative humidity and the presence/absence of oxygen and l-cysteine [322].

Probiotic MPs fabricated by the coacervation of soybean protein concentrate using calcium salts and spray-drying, encapsulating *Lactobacillus plantarum* CECT 220 and *Lactobacillus casei* CECT 475, markedly enhanced both the probiotic viability and the tolerance of tested strains of lactic bacteria against simulated GI fluids compared to current available commercial forms [323]. *Bifidobacterium longum* encapsulated in soy protein isolate–ι-carrageenan (10:1) complex coacervates showed considerably improved viability during storage at 4 °C and pasteurization (85 °C for 5, 10, and 30 min) as well as in vitro dynamic gastric and intestinal digestion. The highest EE of *B. longum* showed coacervates produced at pH 3 [324].

Electrospinning represents a promising method for the entrapment of *Lactobacillus* species into solid delivery systems, where viability correlates with the hydrophobicity and extreme length of lactic acid bacteria [325]. The protection of *Bifidobacterium lactis* and *L. acidophilus encapsulated in SLMPs prepared* by spray-chilling process against GI conditions and improved stability during refrigerated and frozen storage was reported previously also by Pedroso et al. [326], and promising results related to the protection of *L. acidophilus* from GI fluids using cocoa butter as a carrier were obtained as well [327]. *Lactobacillus paracasei* BGP1 and *Lactobacillus rhamnosus* 64 encapsulated in SLMPs covered by a complex of gelatin and GA with sizes ca. 80 μm, which were found to be destabilized only at extreme pH and temperature of 50 °C, achieved higher stability in the presence of salt and showed enhanced resistance in simulated GI conditions, and their viability was not affected by the storage temperature. Moreover, they maintained their functionality and immunomodulating capacity also in an in vivo test in mice [328]. Paula et al. [329] microencapsulated probiotic cells of *L. plantarum* via a dual process: emulsification followed by complex coacervation using gelatin and GA; the particle sizes of microcapsules were 66.07–105.66 ± 3.24 μm, and EE was 97.78%. The viability of encapsulated *L. plantarum* cells was maintained during storage at 8 °C and −18 °C for 45 days, and in vitro in simulated GI conditions 3.22-fold higher viability was observed at 37 °C compared to that estimated for the free cells (50.4% vs. 25%).

SLMPs co-encapsulating *L. acidophilus* (a probiotic) with either inulin or polydextrose (prebiotics) fabricated by spray chilling technology were found to increase the survival rate of the probiotic under exposure to SGF and SIF compared to that of free probiotic cells and the formulations showed good stability when stored refrigerated and frozen with a controlled relative humidity [330]. Lyophilized vegetal BM 297 ATO-inulin lipid-based synbiotic MPs containing *Bifidobacterium longum* LMG 13197 with EE of 82% and particle sizes of 33.4–81.0 μm, which could be evenly distributed in food products and deliver sufficient numbers of viable bacteria, have potential to be used as food additives [331]. *Bifidobacterium* BB-12 microencapsulated in full-fat goat’s milk achieved EE of 97.43% and 94.29% survival rate under simulated GI conditions as well as improved resistance to higher temperatures. The improved survival of encapsulated bifidobacteria exposed to thermal treatments was observed also when prebiotics inulin and/or oligofructose or their combinations with full-fat goat’s milk were used as carrier agents [332].

*L. acidophilus* bacteria encapsulated into nanofibers of polyvinyl alcohol and polyvinylpyrrolidone by electrospinning were characterized with long term stability and maintained viability if they were kept at ≤7 °C [333]. *Lactobacillus* *reuteri* E81 successfully nanoencapsulated into polyvinyl alcohol-based nanofibers with the mean diameter of 381.83 ± 130.69 nm and applied to the surface of fish fillets markedly improved the antioxidant characteristics of fish fillets resulting in a considerable rise in free radical scavenging activity compared to fish fillet samples of the control group; *L.* *reuteri* E81growth in rainbow trout fillets and fatty fish (mackerel) achieved 2.92 and 3.27 − 2.66 log_10_(CFU/g), respectively [334].

In the shadow of all the above facts, the probiotic formulation prepared by encapsulation of *Lactobacillus* sp. to β-glucan [80,319] and O/W NE probiotic consisting of coconut oil and WPC [170], inulin [335], and GA incorporating probiotics could be supposed as superb nanoformulations to support immunity [321].

## 7. Conclusions

Nowadays, when the unhealthy lifestyle associated with lack of exercise and increased consumption of fast foods not only in adults but also in children is beginning to dominate, the number of obese individuals in the developed countries and in the so-called third-world countries is growing rapidly. Even during the current COVID-19 pandemic, it has been shown that in addition to the advanced age (over 80 years) usually associated with polymorbidity, obesity also increases the risk of a severe course of the disease, precisely due to low immunity. Good immunity also makes a significant contribution to reducing the adverse health consequences of increasing environmental pollution and everyday stress. Thus, the importance of a balanced diet containing vitamins, antioxidants, and omega-3 fatty acids in the amount needed for human health is indisputable. In addition, more and more emphasis has recently been placed on the use of probiotics to maintain good health due to the proper composition of the intestinal microflora. Among other things, probiotics are supposed to suppress the side effects of aggressive drugs, such as those used to treat cancer. All of the above agents are able to positively modulate immunity and thus make the human body more resistant to all negative influences. Very often, various foods are fortified with nanoparticles of active ingredients to become so-called smart foods, or nutraceuticals are reformulated into nanonutraceuticals for greater stability, bioavailability, and efficacy. However, it should be noted that the enrichment of industrially produced food products with nanonutraceuticals would be highly desirable even in third-world countries, where people do not have the opportunity to replenish the missing vitamins and other health-promoting substances with commercially produced nutraceutical supplements. However, it is necessary to ensure that all these nutraceutical/nanoscale nutraceutical products are labeled with a health claim confirming that their positive effect was actually observed in the tests performed. Furthermore, it should also be borne in mind that even the best smart food cannot cure an already proven disease and, therefore, does not replace drugs and treatment, but can only help to prevent a disease or contribute to a better tolerability of treatment and increase the effectiveness of drugs. In summary, consuming a diet enriched with nanonutraceuticals enhances immunity and can help people to achieve “a healthy mind in a healthy body”, which contribute significantly to a happy and satisfied life.

## Abbreviations

ALG (alginate); AST (astaxanthin); β-Car (β-carotene); β-CD (β-cyclodextrin); CFU (colony-forming unit); CLPs (colloidal lipid particles); CMC (carboxymethyl cellulose); CMCS (carboxymethyl chitosan); CNC (central nervous system); CoQ10 (Coenzyme Q10); COS (chitooligosaccharide); COVID-19 (coronavirus disease caused by the SARS-CoV-2 virus); CS (chitosan); CUR (curcumin); DHA (docosahexaenoic acid); DO (digestible oil); DPPH (2,2-diphenyl-1-picrylhydrazyl); EE (encapsulation efficiency); EFSA (European Food Safety Authority); EGCG (epigallocatechin-3-gallate); FA (folic acid); FFAs (free fatty acids); FJM (fruit juice-milk); GA (gum arabic); GABA (gamma-aminobutyric acid); GI (gastrointestinal); GIT (gastrointestinal tract); HA (hyaluronic acid); IFNγ (interferon gamma); IL-6 (interleukin-6); IO (indigestible oil); α-LA (α-linolenic acid); LbL (layer-by-layer); LDHs (layered double hydroxides); LNCPs (lipid nanocapsules); MAPK (mitogen activated protein kinase); MCT (medium chain triglycerides); MDX (maltodextrin); MPs (microparticles); Na-ALG (sodium alginate); NaOl (sodium oleate); NE (nanoemulsion); NFC (nanofibrillated cellulose); NLCs (nanostructured lipid carriers); NPs (nanoparticles); ovalbumin (OVA); O/W (oil-in-water); PBMCs (peripheral blood mononuclear cells); PC (phosphatidylcholine); PCR (polymerase chain reaction); PEG (polyethylene glycol); PPI (pea protein isolate); PUFA (polyunsaturated fatty acid); PWP (polymerized whey protein); QR (quercetin); RebA (rebaudioside A); RES (resveratrol); ROS (reactive oxygen species); SGF (simulated gastric fluid); SIF (simulated intestinal fluid); SLNPs (solid lipid nanoparticles); SPI (soy protein isolate); TNFα (tumor necrosis factor alpha); α-Toc (α-tocopherol); W/O (water-in-oil); WP (whey protein); WPC (whey protein concentrate); WPI (whey protein isolate); ZX (zeaxanthin).
